# Comparative transcriptome analysis of the wild-type model apomict *Hieracium praealtum* and its *loss of parthenogenesis* (*lop*) mutant

**DOI:** 10.1186/s12870-018-1423-1

**Published:** 2018-09-24

**Authors:** Sophia Bräuning, Andrew Catanach, Janice M. Lord, Ross Bicknell, Richard C. Macknight

**Affiliations:** 1 Department of Biochemistry, University of Otago, 710 Cumberland St, Dunedin, 9016 New Zealand; 2 Department of Botany, University of Otago, 464 Great King St, Dunedin, 9016 New Zealand; 3 New Zealand Institute for Plant and Food Research, Gerald St, Lincoln, 7608 New Zealand

**Keywords:** Apomixis, Parthenogenesis, Asexual, *Hieracium*, RNA sequencing, Transcriptome

## Abstract

**Background:**

Asexual seed formation (apomixis) has been observed in diverse plant families but is rare in crop plants. The generation of apomictic crops would revolutionize agriculture, as clonal seed production provides a low cost and efficient way to produce hybrid seed. *Hieracium* (Asteraceae) is a model system for studying the molecular components of gametophytic apomixis (asexual seed reproduction).

**Results:**

In this study, a reference transcriptome was produced from apomictic *Hieracium* undergoing the key apomictic events of apomeiosis, parthenogenesis and autonomous endosperm development. In addition, transcriptome sequences from pre-pollination and post-pollination stages were generated from a *loss of parthenogenesis* (*lop*) mutant accession that exhibits loss of parthenogenesis and autonomous endosperm development. The transcriptome is composed of 147,632 contigs, 50% of which were annotated with orthologous genes and their probable function. The transcriptome was used to identify transcripts differentially expressed during apomictic and pollination dependent *(lop)* seed development. Gene Ontology enrichment analysis of differentially expressed transcripts showed that an important difference between apomictic and pollination dependent seed development was the expression of genes relating to epigenetic gene regulation. Genes that mark key developmental stages, i.e. aposporous embryo sac development and seed development, were also identified through their enhanced expression at those stages.

**Conclusion:**

The production of a comprehensive floral reference transcriptome for *Hieracium* provides a valuable resource for research into the molecular basis of apomixis and the identification of the genes underlying the *LOP* locus.

**Electronic supplementary material:**

The online version of this article (10.1186/s12870-018-1423-1) contains supplementary material, which is available to authorized users.

## Background

Apomixis is a type of asexual plant reproduction that bypasses meiosis and fertilization to result in viable seeds that are maternal clones [1]. It is observed in around 400 plant species belonging to 40 families, however very few of these plants are crop species [2]. Introducing the apomixis trait into commercial hybrid seeds would allow fixing hybrid vigor [2]. Because of this agricultural application the ability to transfer or engineer apomixis into crop species is the ultimate goal of many studies of apomixis. As a result, many model plants have been developed to study apomixis [3–10].

Gametophytic apomixis is just one of a range of apomictic developmental pathways. It is characterized by the formation of unreduced embryo sacs either from somatic nucellar cells called aposporous initials (AI) (apospory) or from the Megaspore Mother Cell (MMC) (diplospory), and the subsequent development of the embryo from the unreduced egg cell through parthenogenesis. In some cases, the central cell proliferates autonomously to give rise to the endosperm, however usually it requires fertilization by one sperm cell (pseudogamy) [1]. Most apomicts are pseudogamous, with the exception of a few autonomous apomicts (aposporous), mainly in Asteraceae [11]. The genus *Hieracium* L. (Asteraceae) contains sexual and apomictic species that are aposporous (subgenus *Pilosella*) and diplosporous (subgenus *Hieracium*) [12]. Recently, subgenus *Pilosella* and subgenus *Hieracium* were treated as separate genera, with the aposporous species belonging to genus *Pilosella* and the diplosporous species belonging to the genus *Hieracium* [13]. In this study we still refer to the aposporous group as *Hieracium* subgenus *Pilosella*.

*Hieracium* subgenus *Pilosella* has been developed as a model system to investigate the molecular components of gametophytic apomixis [14]. Various studies have utilized this model to further our understanding of the cytological and molecular events in this group. For instance, the floral staging in relation to gametogenesis, embryogenesis and seed maturation events in sexual and apomictic members of this group was described by Koltunow et al. [9]. Key regulatory processes during seed development were shown to be similar in sexual and apomictic species of *Hieracium* subgenus *Pilosella*; the differences occurred temporally and spatially [15]. Additionally, the genomic regions conferring apomeiosis and parthenogenesis in *H. caespitosum,* also in subgenus *Pilosella*, were identified through a deletion mapping study [16]. These are independent loci termed *LOSS OF APOMEIOSIS* (*LOA*) and *LOSS OF PARTHENOGENESIS* (*LOP*), respectively. *LOA* is involved in the formation of aposporous initials and their subsequent development to make aposporous embryo sacs. Therefore, plants with deleted regions in this locus employ meiosis to produce reduced embryo sacs. These mutants, however, retain the ability of the egg cell and central cell to undergo spontaneous proliferation to give rise to the embryo and endosperm respectively. *LOP* is responsible for parthenogenesis. The deletion of this region is associated with mutants that retain the ability to produce aposporous embryo sacs but require fertilization in order for the embryo and endosperm to develop. These mutants carry the recessive allele (*lop*). Mutants with loss of function in both loci were also found and these mutants employ the sexual pathway to produce seeds [16].

This model system offers unique opportunities to study apomixis. For example, the lack of near identical apomictic and sexual lines has hindered the discovery of transcriptionally active apomixis-linked genes in *Paspalum simplex* [17]. The *Hieracium* model system overcomes this challenge as it encompasses natural apomicts as well as mutants that either completely or partially lost the ability to reproduce asexually. The lack of genomic and transcriptomic reference material for *Hieracium* is another challenge for high throughput analysis of gene expression. We aimed to produce a reference floral transcriptome of *Hieracium*, which will be a useful tool for comparative analysis of gene expression in apomictic, sexual and mutant *Hieracium*.

We carried out a comparative transcriptomics study of an apomictic *Hieracium* accession (R35) and its pollination dependent (*lop*) mutant. This allowed us to compare gene expression during parthenogenesis in the wild type to that which occurs during fertilization-induced embryo development in the mutant accession. This reference transcriptome will be useful in advancing our knowledge about regulatory networks and pathways involved in apomixis. It will also aid the efforts being made to map the genes found in the *LOP* region of *Hieracium*.

## Results

### Determining capitulum stages of R35, *lop138* and A36 that are undergoing embryo development

To profile the expression of genes involved in autonomous seed development, embryo screening was initially used to determine the capitulum stages to be sampled. The wild-type apomictic line, R35, contained mature embryo sacs by stage 6 and had commenced embryogenesis by stage 7. The *lop138* mutant contained mature embryo sacs by stage 6 but was not able to proceed through embryogenesis unless cross-pollinated. The pollinator line, A36, despite being aposporous, took longer to produce mature embryo sacs and initiated embryo development later than R35. These observations allowed the identification of capitulum stages to be sampled in order to produce the transcriptomic data. To investigate transcriptional changes occurring during parthenogenesis and fertilization induced embryo developments, stages before embryogenesis and after embryogenesis for R35 and *lop138* were selected. An “after embryogenesis” stage was also selected for A36 (Table 1).

**Table 1:** Information of developmental events in sampled capitula stages of R35, *lop138* and A36

Species	Accession Id	Parthenogenesis	Sample code	Capitulum stage	Developmental events ^#^
*Hieracium praealtum*	R35	Capable	Ovary_3	3	Tetrads of megaspores present, AIs differentiating
			Ovary_4	4	Meiosis suppression, meiotic tetrads degenerating and AIs enlarging
			Ovary_5	5	Embryo sacs develop from AIs through mitosis
			Ovary_6	6	Mature embryo sacs developed. Some undergo spontaneous embryogenesis.
			Ovary_7	7	Embryo and endosperm development occur in many ovules.
			Ovary_8	8	Ovules contain early to globular embryos
			Ovary_9	9	Ovules contain early to globular embryos
			Ovary10	10	Senescence begins. Ovules contain globular to late globular embryos.
			Wt_E1	5	Embryo sacs develop from AIs through mitosis
			Wt_E2		
			Wt_L1	7	Embryo and endosperm development occur in many ovules.
			Wt_L2		
	l*op138*	Not capable	Lop_E1	9 (not pollinated)	Mature embryo sacs containing quiescent egg cell
			Lop_E2		
			Lop_L1	12 (after pollination)	Ovules contain globular to heart? embryos
			Lop_L2		
*H. aurantiacum*	A36	Capable	Pol_L1	10	Ovules contain globular to heart stage embryos
			Pol_L2		

^#^ Description of developmental events for R35 and A36 was based on [9, 51] and observations from the current study and [16] were used for *lop138*

### RNA isolation from the reproductive tissues sampled

To obtain sufficient RNA for analysis, the reproductive tissue from different staged capitula needed to be manually dissected then pooled. As both the dissection and pooling steps can lead to RNA degradation in the tissue, various solutions were trialed to find a suitable tissue storage and dissection media during the tissue collection process. From these trials it was found that RNAase free water could be used as a dissection medium. Replicated sampling was carried out from the stages listed in Table 1. Multiple ovules and ovaries (50–93) were pooled to isolate ~ 1.4–4.5 μg of total RNA (Additional file 1: Table S1).

### De novo assembly

Sequencing of the prepared cDNA libraries on Illumina’s HiSeq 2000 produced a total of 791,095,876 good quality reads. Trinity assembly yielded a total of 179,195 transcripts belonging to 85,000 components (loci). For each library, 75–81% of reads were mapped back to the reference transcriptome, therefore the transcriptome is representative of all libraries and the sub-sampling of reads to produce a reference transcriptome was deemed to have been effective.

The N50 and average transcript length of the transcriptome were around 500 bp. BUSCO assessment revealed that the transcriptome was incomplete and had some redundancy. The transcriptome was further processed to remove chimeric and redundant transcripts, which resulted in an assembly containing 147,632 transcripts belonging to 74,424 loci. This reduced the number of duplicated BUSCOs by 10%. This reference transcriptome has been deposited at the Shotgun Assembly project DDBJ/EMBL/GenBank under the accession number GEEH00000000.

### Transcriptome annotation

To annotate the proteins potentially encoded in these transcripts, BLASTX searches were performed against two protein databases with an e-value cut-off of 1e-4. The first database contained all NCBI-NR plant protein sequences and BLASTX against it returned similarities for 35,373 of *Hieracium* transcripts (47.5%). The second database was Swiss-Prot, which is manually curated and often supported via experimental or literature evidence. BLASTX against this database identified similarity with 25,377 (34%) of the transcripts. After consolidating the results, a total of 35,436 transcripts (48%) were identified with similarity to proteins in these databases.

**Fig. 1:**
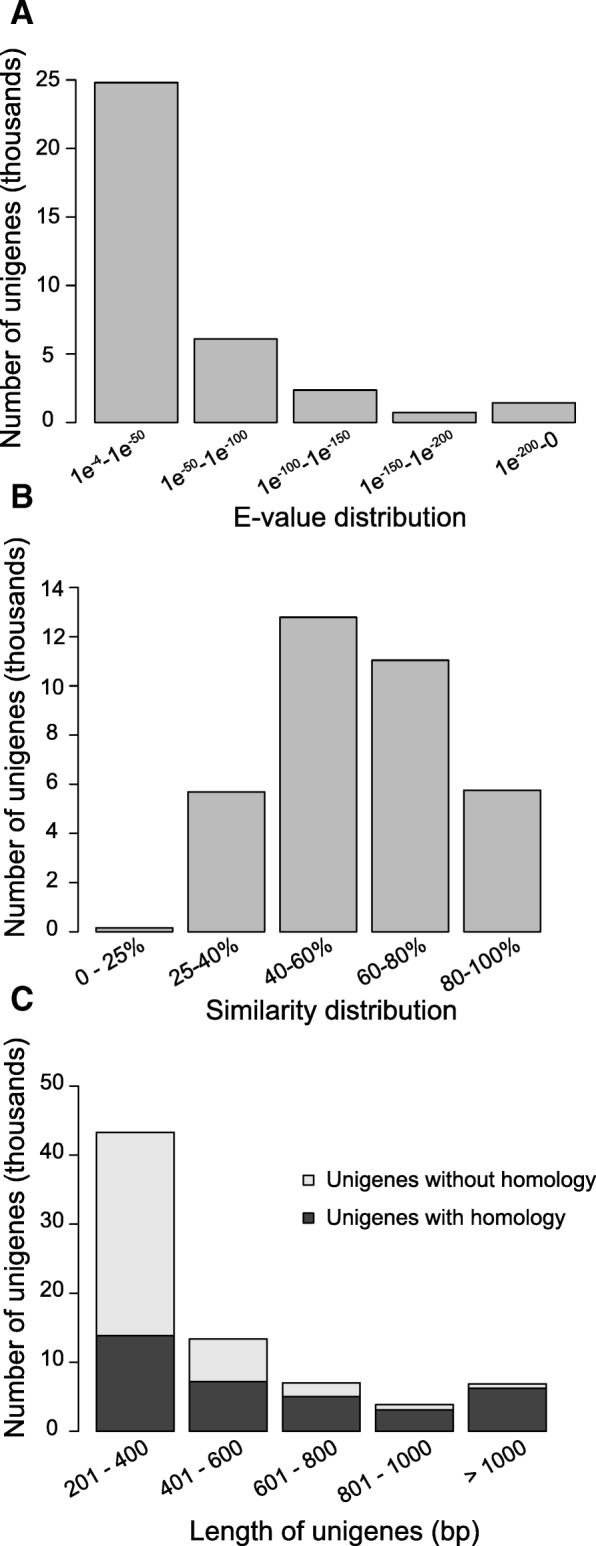
Similarity search result. Putative *Hieracium* genes were searched against NCBI-NR and Swiss-Prot databases. **a** E-value distribution of best BLASTX result of genes (E-value of 1e-4). **b** Similarity distribution of the top BLASTX hit for each gene. **c** Proportion of genes with BLASTX results in relation of the length of genes

The e-value distribution of these BLASTX hits is shown in Fig. 1a and revealed that 30% showed very high similarity, having an e-value lower than 1e-50. Figure 1b displays the distribution of percent similarity of the BLASTX results and indicates that 87% of the matches have over 25% percent identity. 52% of the genes had no match in the databases. As expected, the percentage of genes with similarity increases with gene length (Fig. 1c). For example, 32% of the genes whose length ranged from 201 to 400 bp had database hits, while 90% of those longer than 1000 bp had hits (Fig. 1c). Thus, the reason why more than half of the transcripts lack similarity is likely to be because they do not have a protein coding sequence due to their short length. However, included in this group are transcripts encoding novel proteins and polyadenylated non-coding RNAs.

Transcript abundance across the libraries was estimated by counting reads aligned to each transcript from each library. The raw read counts were normalized and scaled using the TPM and UPC methods of normalization.

### Identification of differentially expressed (DE) transcripts

Capitula of *Hieracium* carry multiple florets that mature asynchronously. As a result, samples from consecutive capitulum stages were expected to represent overlapping developmental stages. To determine the degree of overlap in expressed transcripts between stages, Spearman’s correlation was applied between each consecutive pair of samples. The expression profiles of all samples were positively correlated with that of the prior developmental stage, with the majority having a correlation coefficient above 0.7 (Additional file 1: Table S2). Based on this observation, it was decided that samples produced from consecutive capitulum stages could be combined in order to carry out comparative analysis. Four main comparisons were made to identify expression changes occurring in association with apomictic development and in association with fertilization-induced seed development (Table 2). Additional file 1: Table S3 records the number of DE transcripts identified in these analyses. The results of the comparative analyses conducted using DESeq are presented in Additional files 2, 3, 4, 5.

**Table 2:** Information of compared samples in each pairwise differential expression analysis^a^

DE analysis (Comparison)	Accession	Tissue type	Group A		Group B		Genes targeted	Additional Files containing DE results
			Development	Sample codes	Development	Sample code		
1	R35	Ovules	Mature embryo sac	Wt_E	Spontaneous embryogenesis occurring	Wt_L	Associated with early stage autonomous seed development in R35	Additional file 2
2	R35	Ovules and Ovaries	Mature embryo sac	Wt_E and Ovary_5	Early to late embryos	Ovary_8, Ovary_9 and Ovary_10	Associated with maturing embryo and endosperm in R35	Additional file 3
3	*lop138*	Ovules	Mature embryo sacs	lop_E	Early to late embryos	lop_L	Associated with pollination-induced embryo development	Additional file 4
4	R35 and lop138	Ovules	Mature embryo sacs	Wt_E	Mature embryo sacs	Lop_E	Associated with differences between parthenogenesis capable accessions and the parthenogenesis incapable accession (*lop138*)	Additional file 5
	R35 and lop138	Ovules	Mature embryo sacs	Wt_E	Early to late embryos	Lop_L		
	R35 and lop138	Ovules	Spontaneous embryogenesis occurring	Wt_L	Mature embryo sacs	Lop_E		
	R35 and lop138	Ovules	Spontaneous embryogenesis occurring	Wt_L	Early to late embryos	Lop_L		
	*lop138* and A36	Ovules	Mature embryo sacs	Lop_E	Early to late embryos	Pol_L		
	*lop138* and A36	Ovules	Early to late embryos	Lop_L	Early to late embryos	Pol_L		

^a^ Group A was contrasted to Group B to test for differential expression of each transcript using the DEseq (Anders and Huber 2010) R Bioconductor package

Four comparisons were performed using DE transcripts to discover enriched GO terms (Table 2). Comparison 1 and 2 aimed to identify genes and processes associated with autonomous seed development by comparing samples with mature embryo sacs to samples where spontaneous embryogenesis has occurred (Additional files 2 and 3). GO enrichment analysis of transcripts corresponding to initiation of autonomous seed development (comparison 1) resulted in enrichment of 86 GO terms (Additional file 1: Figure S2). Most of the enriched terms were pertinent to growth and organ morphogenesis, such as regulation of cell growth, regulation of meristem growth, stomatal complex morphogenesis and regulation of organ morphogenesis. GO enrichment analysis of transcripts corresponding to maturing embryos and endosperms in R35 (comparison 2), resulted in 299 enriched GO terms that are summarized in (Additional file 1: Figure S3). In addition to cell and organ growth related terms, this set contained GO terms of embryo and post-embryonic development; such as cotyledon vascular tissue pattern formation, lateral root development, inflorescence morphogenesis, seed coat development, mucilage extrusion from seed coat, seed germination and maintenance of seed dormancy.

Comparison 3 aimed to discover functions enriched due to pollination-induced seed development in the parthenogenesis-incapable accession (*lop138*). This resulted in 384 enriched GO terms (Additional file 4) belonging to the broad GO term categories (Additional file 1: Figure S4). Most terms enriched in this set were also enriched in DE sets of R35 (Additional file 1: Figures S2 & S3). However, in the ‘Reproduction’ category, GO terms associated with sexual reproduction were enriched for *lop138* only, which indicated that in the absence of the *LOP* locus the aposporous embryo sac was functionally equivalent to a sexual embryo sac. The specific ‘sexual reproduction’ terms were; regulation of double fertilization forming a zygote and endosperm, regulation of induction of conjugation upon nitrogen starvation, pre-meiotic DNA replication and megasporogenesis. In addition, the GO term ‘epigenetic regulation of gene expression’ was enriched for *lop138* but not for R35.

Comparison 4 aimed to discover functions associated with differences between the parthenogenesis-capable accessions and the parthenogenesis-incapable accession (*lop138*). A total of 16,530 sequences that were DE in at least one pairwise comparison (*lop138* vs. apomictic accessions) were analyzed to identify enriched GO terms (Table 2). This resulted in the enrichment of 518 GO terms (Additional file 5), belonging to the GO categories detailed in (Additional file 1: Figure S5). The enriched GO categories were similar to the GO categories enriched in the DE sets of R35 (resulting from comparison 2, Additional file 3) and *lop138* (resulting from comparison 3, Additional file 4).

The GO analyses were performed using all the transcripts in the reference datasets that had GO annotations (Additional file 6). A limitation of the GO analysis is that only 48% of the transcriptome was annotated. Therefore, there are a large number of DE transcripts without GO annotation (some of which have BLAST hits but no associated GO annotation) which could not be included in the GO analyses. These transcripts are listed in Additional file 7 and (Additional file 1: Table S4) provides a summary of the number of DE transcripts without GO terms in the different categories.

In summary, all DE sets resulting from the four comparisons shared enriched GO terms. The main differences between the DE sets were that terms associated with epigenetic regulation of gene expression and sexual reproduction were enriched for *lop138* but not for R35. This observation indicated that a lot of transcripts involved in epigenetic regulation and other sexual reproduction processes were differentially expressed during pollination-induced seed development in *lop138* but not in R35, which undergoes autonomous seed development.

### Global expression patterns of differentially expressed transcripts

#### Epigenetic regulation

**Fig. 2:**
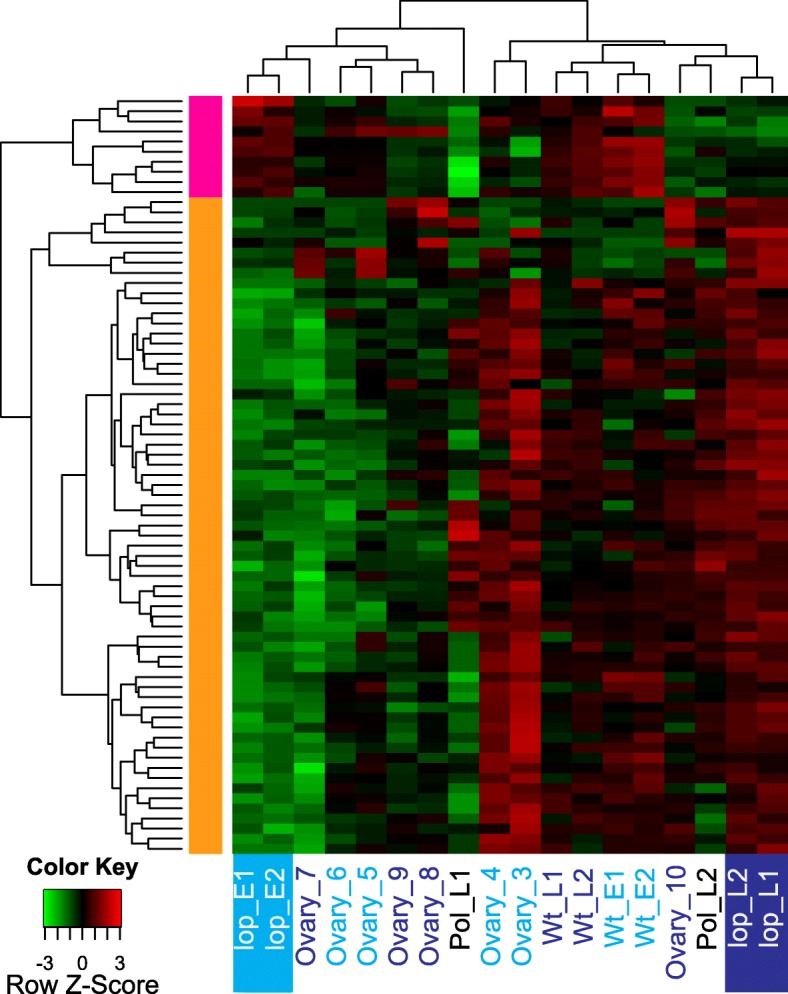
Expression patterns of DE transcripts associated with epigenetic regulation. Hierarchically clustered expression heatmap with rows represent each transcript and relative expression (low in green and high in red) in the samples as indicated. Pre-embryo stages are indicated in light blue and post-embryo stages in dark blue text; the *lop* mutant pre-pollination (*lop* E1, E2) and post pollination (*lop* L1 and L2) are also highlighted)

Hierarchical clustering was used to explore global expression changes of DE transcripts annotated with epigenetic regulation of gene expression (Fig. 2; Additional file 8). The clustering analysis highlighted that most of the transcripts that were up-regulated during embryo development in *lop138* (Fig. 2) were associated with DNA methylation and chromatin silencing functions (Additional file 8). Many of the transcripts associated with epigenetic regulation were up-regulated in *lop138*, showing with a high expression at early megagametogenesis stages of R35 (Ovary_3, Ovary_4) and continuing to show some level of expression in the ovules at stage 5 (Wt_E) and stage 7 (Wt_L) (Fig. 2). This suggests that during autonomous seed development in R35, genes putatively involved in DNA methylation and chromatin silencing were expressed but did not show dramatic changes in expression in response to embryo and endosperm development. This could relate to the absence of a paternal genome in the developing seeds of R35, which may require less restructuring of the epigenome state in the central cell and early endosperm. In sexual plants such as maize, rice and *Arabidopsis*, demethylation of genomic DNA occurs prior to fertilization of the central cell [18–21]. Demethylation allows transcription of transposable elements (TEs) and the production of small interfering RNA (siRNA) that can be used as mobile elements to recognize and silence TEs in the embryo post-fertilization. As the deletion of the *LOP* locus restores sexuality in the aposporous embryo sac of *lop138*, global demethylation may have occurred in the unpollinated ovules of *lop138*. Following pollination, TEs in the embryo of *lop138* may be epigenetically silenced leading to up-regulation of many putative genes involved in epigenetic regulation.

Relatively few transcripts associated with epigenetic regulation were up-regulated in the pre-pollination sample of *lop138*. In R35, these transcripts showed high expression in both pre-embryo (Wt_E) and post-embryo (Wt_L) samples (group 1, Fig. 2). Many of these transcripts, belonging to group 1 in Fig. 2, encode putative orthologs of argonaute 4 (AGO4). Argonaute (AGO) proteins are core components of small RNA-induced silencing pathways; they can act in the post-transcriptional gene silencing (PTGS) pathway and the transcriptional gene silencing (TGS) pathway.

#### Heterochrony

**Fig. 3:**
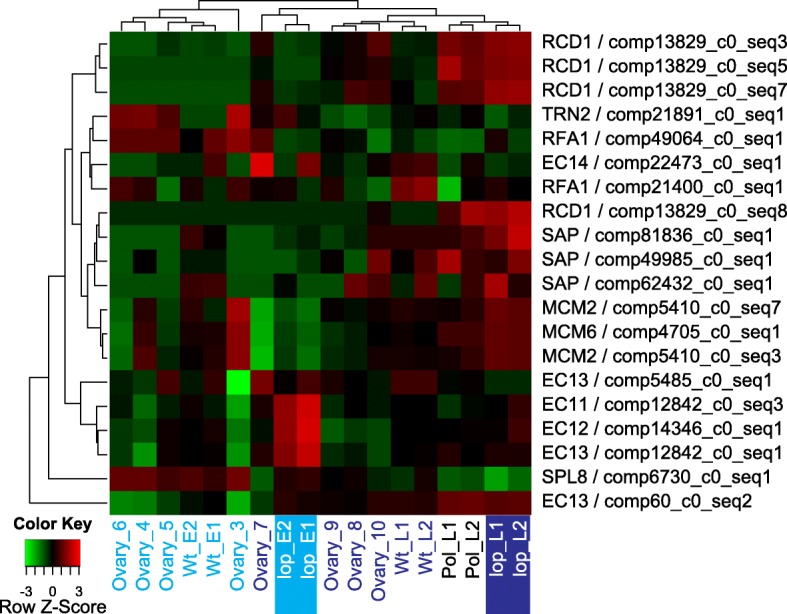
Expression pattern of DE transcripts (*lop138* pre- vs post-pollination) associated with the GO category ‘reproduction’. Hierarchically clustered expression heatmap with rows represent each transcript (labeled by the putative protein and the transcript ID) and relative expression (low in green and high in red) in the samples as indicated. Pre-embryo stages are indicated in light blue and post-embryo stages in dark blue text; the *lop* mutant pre-pollination (*lop* E1, E2) and post pollination (*lop* L1 and L2) are also highlighted)

The results from the differential expression and GO term enrichment analysis did not identify mechanisms that were unique to the parthenogenesis-capable wild type R35. This suggested that the differences between apomictic and *lop138* seed development were likely to be spatio-temporal. Many studies propose apomixis to be the result of deregulation of the sexual reproduction pathway; therefore, during apomixis genes and regulatory mechanisms of sexual reproduction are employed in a heterochronic fashion [14, 15, 22–24]. To explore heterochrony in *Hieracium*, global expression patterns of the differentially expressed transcripts with the GO term category ‘reproduction’ were investigated (Fig. 3). Within this category, the GO term ‘megasporogenesis’ was enriched due to the expression of transcripts predicted to code proteins similar to the transcriptional regulator STERILE APETALA (SAP), squamosa promoter-binding-like protein 8 (SPL8) and protein TORNADO 2 (TRN2) (Fig. 3). In Arabidopsis *SPL8*, *SAP* and *TRN2* have regulatory roles that are important for initiation of female gametogenesis and other developmental processes [25]. Therefore, if putative orthologs of *SPL8*, *SAP* and *TRN2* perform similar tasks in *Hieracium* as in *Arabidopsis*, these genes can be expected to be more active during stages involving female gametogenesis, which in wild type *Hieracium* is complete around stage 6 (Table 3).

**Table 3:** Summary of expression pattern of transcripts differentially expressed in *lop138*, putatively encoding for proteins involved in reproduction

GO term	Proteins	Transcript ID	Stage showing high expression in R35	Stage showing high expression in lop138
Megasporogenesis	SAP	Comp81836_c0_seq1	Pre-embryo (stage 5) and post-embryo (stages 7 and 10)	Post-pollination
	SAP	Comp49985_c0_seq1	Post-embryo (stages 7 and 10)	Post-pollination
	SAP	Comp62432_c0_seq1	Pre-embryo (stage 5) and post-embryo (stages 7, 8 and 10)	Post-pollination
	SPL8	Comp6730_c0_seq1	Pre-embryo (stages 3–6)	Pre-pollination
	TRN2	Comp21891_c0_seq1	Pre-embryo (stages 3–6)	Pre-pollination
Pre-meiotic DNA replication	MCM2	Comp5410_c0_seq7	Pre-embryo (stages 3 and 4)	Post-pollination
	MCM2	Comp5410_c0_seq3	Pre-embryo (stages 3 and 4)	Post-pollination
	MCM6	Comp4705_c0_seq1	Pre-embryo (stages 3 and 4)	Post-pollination
	RAF1	Comp49064_c0_seq1	Pre-embryo and post-embryo (stages 3–7)	Unchanged
Regulation of induction of conjugation upon nitrogen starvation	RCD1	Comp13829_c0_seq3	Post-embryo (stages 7–10) (three sequences)	Post-pollination (four sequences)
	RCD1	Comp13829_c0_seq5	Post-embryo (stages 7–10)	Post-pollination
	RCD1	Comp13829_c0_seq7	Post-embryo (stages 7–10)	Post-pollination
	RCD1	Comp13829_c0_seq8	unchanged	Post-pollination
Regulation of double fertilization forming a zygote and endosperm	EC1.4	Comp22473_c0_seq1	Pre-embryo (stage 5) and Post-embryo (stage 7)	Pre-pollination
	EC1.3	Comp5485_c0_seq1	Pre-embryo (stage 5) and Post-embryo (stage 7)	Pre-pollination
	EC1.1	Comp12842_c0_seq3	Pre-embryo (stage 5) and Post-embryo (stage 7)	Pre-pollination
	EC1.2	Comp14346_c0_seq1	Pre-embryo (stage 5)	Pre-pollination
	EC1.3	Comp12842_c0_seq1	Pre-embryo (stage 5) and Post-embryo (stage 7)	Pre-pollination
	EC1.3	Comp60_c0_seq2	Post-embryo (stages 8–10)	Post-pollination

The reproduction associated GO term ‘pre-meiotic DNA replication’ was enriched due to putative orthologs of DNA replication licensing factor *mcm2–7* (*MCM2–7*) complex and replication factor A protein 1 (*RFA1*) (Table 3). The MCM2–7 complex proteins plays a role that ensures replication occurs once per cell cycle [26]. *RFA1* is conserved in all eukaryotes and is required for DNA replication, repair and recombination [27]. Based on their roles during cell division, the expression of the putative *MCM2–7* complex and *RFA1* can be expected to increase during stages undergoing cellular proliferation. In *lop138*, cell division stops after the embryo sac matures, therefore expression of genes involved in cell division can be expected to increase in the post-pollination stage as cell division resumes during embryogenesis. In R35, the mature embryo sac proceeds through spontaneous embryogenesis; therefore, expression of genes involved in cell division may not differ dramatically between the mature embryo sac and the post-embryo developmental stage. Putative orthologs of *MCM2 and MCM6* were identified as up-regulated in *lop138* post-pollination but not in R35 post-embryo samples (Fig. 3; Additional file 1: Tables S3, S4 and S5). Although not DE in R35 post-embryo samples relative to pre-embryo samples, putative *MCM2* and *MCM6* exhibited relatively high expression in stage 3 and 4 samples of R35 (Fig. 3). In R35, putative *RFA1* expression was high during stages undergoing female gamete development (3–5) and early embryo development (stages 6–7). In *lop138*, putative *RFA1* expression did not change during embryo development (Fig. 3).

The reproduction associated GO term ‘regulation of induction of conjugation upon nitrogen starvation’ was enriched due to transcripts predicted to encode proteins with similarity to cell differentiation protein RCD1, which is involved in the onset of sexual reproduction through its differentiation controlling function in yeast [28]. Four transcripts predicted to encode RCD1 were up-regulated in the post-pollination sample of *lop138* and also showed high expression in the post-embryo sample of A36 (Fig. 3). Compared to pre-embryo stages (3–5) of R35, three of these putative *RCD1* orthologs also showed increased expression in post-embryo stages (7–10) of R35 (Fig. 3). The GO term ‘regulation of double fertilization forming a zygote and endosperm’ was enriched because of transcripts that are likely to encode proteins similar to egg cell-secreted (EC1) proteins of *Arabidopsis*. In *Arabidopsis*, five functionally redundant *EC1*-like genes (*EC1.1*, *EC1.2*, *EC1.3*, *EC1.4* and *EC1.5*) were specifically expressed in the egg cell prior to fertilization and regulated gamete interaction during double fertilization [29]. Based on *EC1*’s function and expression patterns in *Arabidopsis*, putative *EC1* was expected to be active in *lop138*, which employs double fertilization during seed development. While six putative *EC1* orthologs were identified as DE in l*op138*, *EC1* orthologs were not identified as DE in R35 (Additional files 2, 3, 4, 5). Although not DE in R35, putative *EC1* orthologs showed some level of expression in both pre-embryo (Wt_E) and post-embryo (Wt_L) ovule samples as well as in the post-embryo A36 sample (Fig. 3). Up-regulation of one of the putative *EC1* orthologs in post-pollination sample of *lop138* may indicate that some egg cells had not yet initiated embryogenesis.

**Fig. 4:**
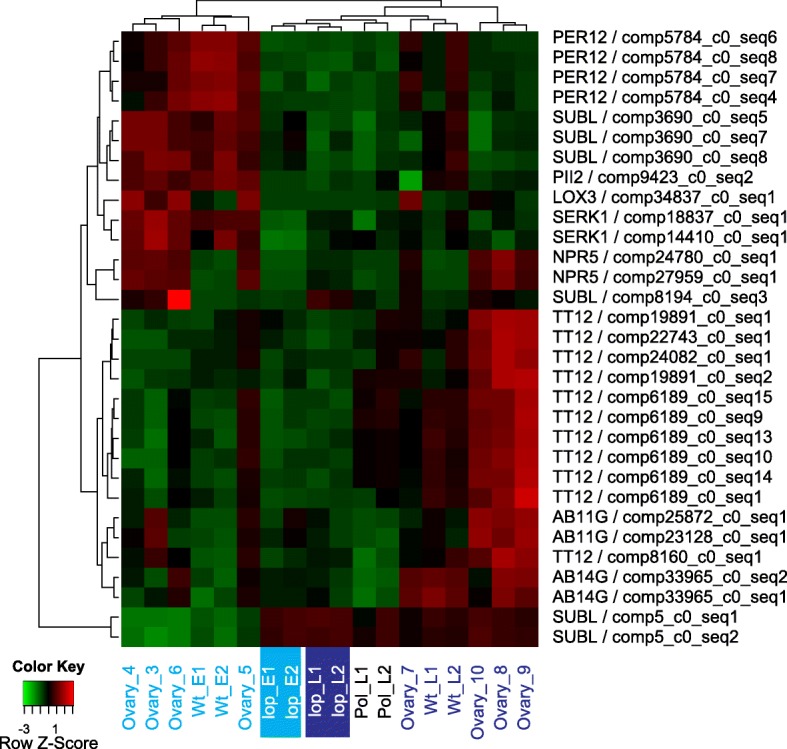
Expression patterns of DE transcripts (*lop138* vs WT) associated with the GO category ‘reproduction’. Hierarchically clustered expression heatmap with rows represent each transcript (labeled by the putative protein and the transcript ID) and relative expression (low in green and high in red) in the samples as indicated. Pre-embryo stages are indicated in light blue and post-embryo stages in dark blue text; the *lop* mutant pre-pollination (*lop* E1, E2) and post pollination (*lop* L1 and L2) are also highlighted)

For transcripts that were DE in R35 relative to *lop138* samples, the ‘reproduction’ category contained the GO terms ‘cotyledon vascular tissue pattern formation’, ‘maintenance of seed dormancy’, ‘mucilage extrusion from seed coat’ and ‘reproductive structure development’. Figure 4 shows the expression patterns of transcripts associated with these terms. Transcripts associated with floral structure development were mostly up-regulated in the R35 pre-embryo samples and putatively encode ABC transporter B family member 19 (AB19B), peroxidase 12 (PER12), piriformospora indica-insensitive protein 2 (PII-2), somatic embryogenesis receptor kinase 1 (SERK 1), regulatory protein NPR5 (NPR5) and lipoxygenase 3 (LOX3) (Fig. 4). Many of these proteins play a role in plant development through their involvement in auxin transport (AB19B) [30], response to biotic and abiotic stress (perioxidases) [31], mediating symbiotic relationship of fungus and host to promote growth and seed production (PII-2) [32], promoting leaf and floral meristem fate and determinacy through positive regulation of adaxial-abaxial polarity genes such as *YAB1* and *YAB3* (NPR5) [33, 34] and involvement of LOXs in plant growth, pest resistance, senescence or responses to wounding [35, 36]. Most notably *SERK* genes code for a transmembrane protein involved in signal transduction and have been associated with somatic embryogenesis and apomixis and function in plant response to biotic and abiotic stimuli in a number of plant species [37]. Therefore up-regulation of putative *SERK1* transcripts in pre-embryo samples of R35 may be an indication that this gene plays a role in inducing parthenogenesis in R35. Transcripts associated with mucilage extrusion from the seed coat were putative orthologs of subtilisin-like protease (SUBL). More transcripts with similarity to SUBL were up-regulated in the pre-embryo samples of R35 (Fig. 4).

Putative orthologs of ABC transporter G family member 11 (AB11G) and ABC transporter G family member 14 (AB14G) were associated with cotyledon vascular tissue pattern formation. These transcripts were up-regulated in the post-embryo samples of R35 (Fig. 4). Transcripts associated with maintenance of seed dormancy were putative orthologs of *TRANSPARENT TESTA 12* (*TT12*) and were up-regulated in the post-embryo samples of R35 (Fig. 4). The expression patterns observed for transcripts likely encoding AB11G, AB14G and TT12 fit with their associated function in cotyledon tissue formation and seed maturation as seed development progressed in R35.

#### Embryo patterning genes and polar auxin transport

**Fig. 5:**
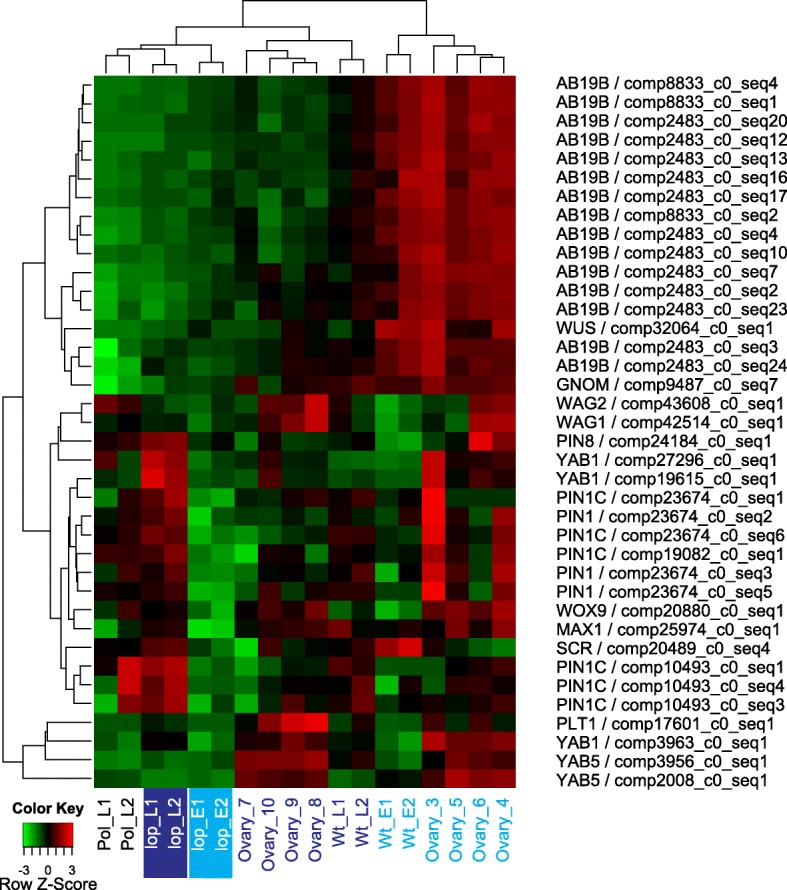
Expression patterns of DE transcripts associated with auxin transport and embryo development. Hierarchically clustered expression heatmap with rows represent each transcript (labeled by the putative protein and the transcript ID) and relative expression (low in green and high in red) in the samples as indicated. Pre-embryo stages are indicated in light blue and post-embryo stages in dark blue text; the *lop* mutant pre-pollination (*lop* E1, E2) and post pollination (*lop* L1 and L2) are also highlighted)

During early embryogenesis, asymmetrical division in the zygote establishes the apical-basal axis of the embryo. While the basal cell transports the growth regulator hormone auxin, the apical cell responds to auxin and this apical-basal gradient of auxin triggers the specification of apical embryo structures. Auxin carriers such as PIN-FORMED1 (PIN1) and their polar localization play an important role in the specification of apical-basal pattern formation of early embryo and root formation. The polar localization of auxin carriers is dependent on directed vesicle trafficking, which is regulated by the ARF guanine-nucleotide exchange factor GNOM [38]. A *Hieracium* transcript putatively encoding GNOM was up-regulated in the pre-embryo stages of R35 (Fig. 5). Many transcripts associated with auxin transport were also up-regulated in the pre-embryo stages of R35. These were AB19B, serine/threonine-protein kinases WAG1 and WAG2, MAX1 and PIN proteins. Most of the PIN proteins were also up-regulated in the post-pollination sample of *lop138* (Fig. 5). AB19B is involved in auxin transport and together with PIN proteins, establishes the polar auxin concentration gradient [30]. WAG1 and WAG2 are also involved in the regulation of auxin signaling through enhancing PIN-mediated polar auxin transport [39]. MAX genes are proposed to inhibit axillary bud growth by affecting PIN-mediated auxin transport in the stem [40]. Up-regulation of transcripts involved in auxin transport in the pre-embryo stages of R35 indicates that the phytohormone auxin and its concentration gradient in ovaries of *Hieracium* may be an important cue for induction of autonomous embryo development.

*WUSCHEL* (*WUS*) regulates stem cell formation and maintenance [41] and in *Arabidopsis* is first expressed at the 16-cell stage of embryogenesis in cells that will form the shoot apical meristem, or SAM [42]. A plant specific homeodomain transcription factor, *WUSCHEL-RELATED HOMEOBOX9* (*WOX9*), promotes stem-cell fate by positively regulating the expression of *WUSCHEL (WUS)* in the SAM [43]. In R35, putative *WUS* homolog expression was high in pre-embryo stages (Fig. 5), which could indicate that an earlier induction of *WUS* in apomictic ovaries of *Hieracium* promotes stem cell identity and maintenance, leading to precocious somatic embryogenesis. The putative homolog of *WOX9* also showed up-regulation in pre-embryo and post-embryo ovaries of R35, which further indicates that stem cell specification may begin during early ovary development prior to the morphological observation of the apomictic embryo. Other embryo developmental patterning genes, such as *SCARECROW* (*SCR*) [44] and *YABBY* genes [45] were also identified (Fig. 5).

#### Endosperm development

While the endosperm develops autonomously in R35, it requires fertilization in *lop138*. Transcripts predicted to encode proteins with similarity to proteins that play critical roles in endosperm development in other plants such as members of the PcG complex (FIS1/MEA, FIS2, FIS3/FIE, RBR and MSI1), were investigated to find out if they differentially express in R35 or *lop138*. Expressions of three members of FIS complex (FIE, RBR and MSI1) were supported by the high percent similarities between the respective *Arabidopsis* protein sequences and the predicted *Hieracium* peptides (above 70% identity). However, only three putative *MSI1* orthologs were up-regulated during embryo development in *lop138.* There was no evidence of expression of *MEA* and *FIS2* in *Hieracium*, however expression of *SWINGER* and *CLF* was supported. Table 4 lists sequences predicted to encode PcG proteins and their DE status.

**Table 4:** Transcripts predicted to encode Polycomb Group (PcG) proteins based on top BLASTX hits to *Arabidopsis* proteins

Transcript ID	Transcript length (bp)	Putative protein	% identity	e-value	Differentially expressed^a^
Comp8804_c0_seq1	1271	FIE	74	0	NO
Comp7474_c0_seq1	743	RBR	99.3	7E^−99^	NO
Comp7474_c0_seq4	281	RBR	100	1E^−57^	NO
Comp7474_c0_seq5	266	RBR	98.86	2E^−51^	NO
Comp7474_c0_seq6	252	RBR	100	3E^−47^	NO
Comp7474_c0_seq7	222	RBR	100	5E^−46^	NO
Comp22708_c0_seq1	945	MSI1	62	8E^−98^	Up-regulated in *lop*138 post-pollination relative to *lop*138 pre-pollination
Comp22708_c0_seq2	339	MSI1	74.11	9E^−59^	Up-regulated in *lop*138 post-pollination relative to *lop138* pre-pollination
Comp20599_c0_seq1	355	MSI1	75	7E^−10^	Up-regulated in *lop*138 post-pollination relative to *lop*138 pre-pollination
Comp2957_c0_seq1	1637	MSI1	92.62	0	NO
Comp76669_c0_seq1	280	MSI1	89.25	1E^*−53*^	NO
Comp7074_c0_seq1	2434	EZA1/SWINGER	75.55	0	NO
Comp12352_c0_seq1	2455	CLF	68.98	8E^− 157^	NO
Comp16067_c0_seq1	357	CLF	38.83	1E^−10^	NO
Comp16067_c0_seq6	233	CLF	64.47	3E^−22^	NO
Comp16067_c0_seq7	231	CLF	81.58	6E^−20^	NO
Comp16067_c0_seq8	218	CLF	53.85	2E^−14^	NO
Comp16067_c0_seq9	216	CLF	60.32	1E^−13^	NO
Comp16067_c0_seq10	204	CLF	76.47	1E^−31^	NO
Comp16067_c0_seq11	202	CLF	56.52	7E^−05^	NO

^a^ The ‘Differentially expressed’ column indicates whether the transcript was DE at an adjusted *p*-value of 0.1 in any of the DE analyses performed. The DE analyses were; R35 pre-embryo vs. post-embryo, *lop138* pre-pollination vs. post-pollination, *lop138* vs. R35 and *lop138* vs. A36. More detail on the DE analyses can be found in Table 1

**Fig. 6:**
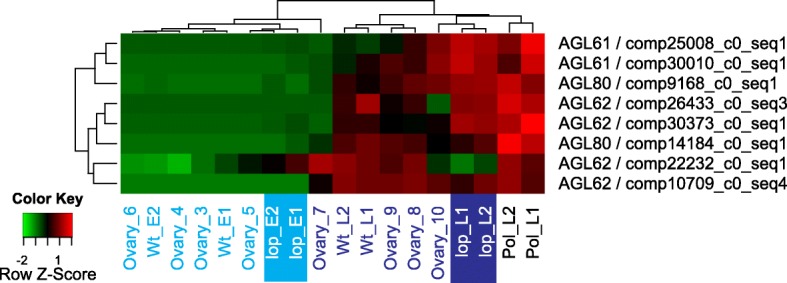
Expression patterns of DE transcripts homologous to *AGL61, AGL62* and *AGL80*. Hierarchically clustered expression heatmap with rows represent each transcript (labeled by the putative protein and the transcript ID) and relative expression (low in green and high in red) in the samples as indicated. Pre-embryo stages are indicated in light blue and post-embryo stages in dark blue text; the *lop* mutant pre-pollination (*lop* E1, E2) and post pollination (*lop* L1 and L2) are also highlighted)

In *Arabidopsis*, Agamous-like MADS-box proteins (AGL61, AGL62 and AGL80) are required for central cell and endosperm development and are exclusively expressed in the central cell and uncellularized endosperm [46–48]. To gain insight into the expression of genes with known domains of expression in *Arabidopsis*, all DE transcripts putatively encoding AGL61, AGL62 or AGL80 proteins (Additional file 1: Table S5) were heat mapped (Fig. 6). The majority of the transcripts showed high expression at post-embryo stages of *lop138*, R35 (stages 7–10) and A36, which reflects the syncytial phase of endosperm development.

### Genes showing enhanced expression during a developmental stage

The transcriptome produced for this study included consecutive developmental stages, ranging from megagametogenesis to embryogenesis and seed maturation processes in the ovary (Ovary_3 – Ovary_10). The ovary data set is therefore ideal for two purposes; 1) to investigate unique processes occurring at each capitulum stage, which may lead to the identification of processes contributing to initiation and progression of apomixis in R35, and 2) to identify molecular markers of key developmental stages.

**Fig. 7:**
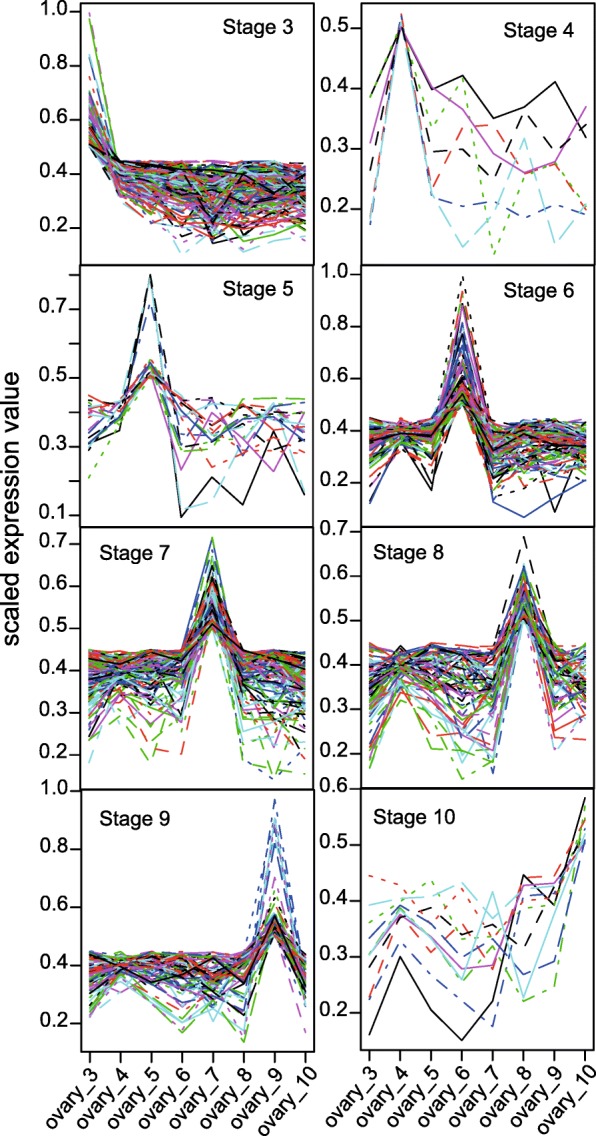
Capitulum stage-enhanced gene expression. Vertical axis is the UPC value and the horizontal axis shows the R35 ovary sample codes, the numbers in the sample code represent the capitulum stage sampled. The UPC values were plotted across the capitulum stages in order to select those that showed expression peaks in a capitulum stage (> 0.5 UPC)

To identify developmental stage markers for *Hieracium*, the expression of each gene was represented by a UPC value of 0 (inactive) to 1 (active) [49]. To simplify the analysis, only the longest transcript of each component was chosen as the representative for that component (gene). The UPC values were plotted across the time points in order to select those that showed expression peaks in a capitulum stage (0.5 being the cutoff) (Fig. 7). While stage 3 showed the highest level of genes showing enhanced activity, stages 4, 5 and 10 exhibited low numbers of genes showing enhanced activity at these capitulum stages. Additional file 9 lists BLASTX results and read counts supporting expression of *Hieracium* genes showing stage-enhanced activity.

KOBAS 2.0 [50] was used to identify enriched pathways and GO terms in the gene sets showing stage-enhanced expression. Additional file 1: Table S6 presents enriched pathways for the stage-enhanced expression data sets. Capitulum stage-enhanced gene expression analysis of the ovary data set of R35 revealed that capitulum stage 3, in which AIs are differentiating in the vicinity of the megaspore tetrads, was enriched in pathways involved in biosynthesis and degradation of IAA. IAA is the most common naturally occurring plant hormone of the auxin class and is involved in various aspects of plant growth and development (Frick and Strader, 2018). Capitulum stages in which the aposporous embryo sac develops and matures (stages 4 and 5), did not present many genes exhibiting enhanced expression during these stages, nor did they have enriched functions. Ovaries from capitula at stage 6 and 7, in which parthenogenesis is expected to initiate, had a relatively high number of genes with enhanced expression, however these gene sets did not result in enrichment of a reproduction pathway term (Additional file 1: Table S6).

### Developmental stage marker genes of Hieracium

To identify reliable developmental stage markers for *H. praealtum*, a UPC value of 0.5 was used as a cutoff to identify genes active during specific developmental stages. Developmental stage markers were identified for: 1) stages undergoing aposporous embryo sac development in R35, i.e. stages 3 to 6 (Additional file 10 A); 2) stages undergoing embryo development in R35 and *lop138*, i.e. stages 7–10 in R35 and post-pollination stage for *lop138* (Additional file 10 B); 3) Markers for apomictic R35; i.e. transcripts active at any stage in R35 but not active in one or both stages of *lop138* (pre-pollination and post-pollination stages) (Additional file 10 C).

The transcripts were identified as potential markers for aposporous embryo sac development stages (Additional file 1: Table S7). Many of these were predicted to encode proteins playing a role in lipid transfer or lipid metabolism and three transcripts that showed similarity to the transcription factor genes, *GATA18*, *IAA32* and *bHLH61,* were identified (Additional file 1: Table S7). The transcripts identified as potential markers for stages undergoing embryo development in R35, *lop138* and A36 encoded transcription factors known to function during seed development in *Arabidopsis*, such as MADS-box transcription factor *PHERES1*, Agamous-like MADS-box protein AGL61 and Nuclear transcription factor Y subunit B-6 and B-9 (Additional file 1: Table S8). Additional file 1: Table S9 lists potential markers of apomictic R35, i.e. transcripts active in one or more developmental stage of R35 but not active in one or both stage of *lop138*.

## Discussion

*Hieracium* subgenus *Pilosella* contains both apomictic and sexual species, which have been developed into model systems for studying molecular and genetic components of apomixis [9]. Previous work used the aposporous *H. praealtum* (R35) for a deletion-based discovery of the two independent dominant loci conferring the key components of apomixes [16]. The locus *LOSS OF APOMEIOSIS* (*LOA*) was found to be responsible for avoidance of meiosis during embryo sac development and mutants missing this locus reverted to forming meiotically reduced embryo sacs. The locus *LOSS OF PARTHENOGENESIS* (*LOP*) was found necessary for parthenogenesis and autonomous endosperm development and mutants missing this locus reverted to the requirement of fertilization for embryo and endosperm development [16]. The availability of deletion mutants that reverted to the sexual mode of reproduction within the apomictic R35 background makes R35 and derived deletion mutants attractive for transcriptome studies. To facilitate discovery of transcriptional differences between apomixis and amphimixis, this study set the goal of producing a reproductive tissue reference transcriptome for subgenus *Pilosella*. R35, *lop* mutant accession (*lop138*) and an additional aposporous species belonging to subgenus *Pilosella*, *H. auranticacum* (A36), were used to produce the reference transcriptome.

To produce the transcriptome, we first needed to develop protocols for sampling reproductive tissue of *Hieracium* that can yield good quality RNA for sequencing. Here, a manual dissection procedure was developed which involved the use of RNase-free water for submerging florets during dissection and the use of an ammonium salt solution for storing collected ovules until RNA could be extracted. This procedure is novel in *Hieracium* and provides a valuable template for further work involving *Hieracium* and other Asteraceae species. The procedure for manually dissecting whole ovaries was relatively quick, thus ovaries were directly collected into RNA extraction buffer then processed to obtain high quality RNA from ovaries and ovules of *Hieracium* (Additional file 1: Table S1). Rabiger et al. [51] also reported manually dissecting *Hieracium* ovaries into liquid nitrogen then extracting RNA, which confirms the relative ease of sampling ovaries.

Developmental stages encompassing aposporous embryo sacs to autonomously developing seeds were sampled from R35 and A36. Developmental stages prior to pollination and post pollination were sampled from the mutant *lop138*. A reference transcriptome from all of these developmental stages was generated. This reference transcriptome is the first de novo transcriptome for *Hieracium* that combines data from all key events of apomixis (aposporous embryo sac development, parthenogenesis and autonomous endosperm developments) and partially sexual events (i.e. pollination-induced embryo and endosperm developments). The reference transcriptome has been deposited at the Shotgun Assembly project DDBJ/EMBL/GenBank under the accession GEEH00000000.

The transcriptome was annotated through extensive BLAST searches and GO term mapping. Recently, ovary transcriptomes from two early apomictic events of *H. praealtum* (MMC and Functional Megaspore development stages) have been released by [51]. The reference transcriptome presented here provides additional resources for early apomictic events and is relevant to the key apomictic events of parthenogenesis and autonomous endosperm development, which makes it a valuable contribution to apomixis research. The representation of data from various stages and plant accessions (apomictic R35 and A36, and partially sexual *lop138*) makes the transcriptome useful for comparative analysis of apomictic and sexual *Hieracium.*

Differential expression analyses were performed to discover functional enrichment in the differentially expressed sequences of the apomictic and *lop138* accessions. This was accomplished by identifying gene expression changes in response to pollination-independent seed development in R35 and the changes occurring in response to pollination-induced seed development in the mutant accession *lop138* (Additional files 2, 3, 4, 5). Pollination-induced seed development in *lop138* resulted in more transcriptional changes than pollination-independent seed development in R35. However, GO term enrichment analysis of the differentially expressed transcripts for R35 and *lop138* respectively, revealed that the two differentially expressed groups of transcripts shared many enriched GO term categories. There were few GO term categories enriched in *lop138* only, one of which was epigenetic regulation of gene expression. The other GO categories enriched in *lop138* only were behavior, cell cycle and DNA metabolic process. Samples of *lop138* were also compared to samples of the apomictic R35 and A36 accessions in order to discover enriched functions associated with differences between *lop138* and the apomictic R35 and A36 (Additional files 4 and 5). Transcripts differentially expressed in *lop138* when compared to the apomictic accessions, resulted in enrichment of many GO term categories that were also shared by the classes of transcripts differentially expressed during seed development in *lop138* and in R35 (Additional file 1: Figure S5). This highlights that apomictic and sexual seed developmental pathway display many common features. Many studies have suggested that deregulation of the sexual reproduction pathway is the likely cause of apomixis, hence apomixis and sexual reproduction are expected to share key components [15, 24].

Orthologs of molecular signals necessary for sexual reproduction, such as *EC1* and *RCD1* were identified as differentially expressed during fertilization-induced seed development in *lop138* and their expression was also detected in R35 and A36 (Fig. 3). In *Arabidopsis*, EC1 proteins regulate gamete interaction during double fertilization, and are specifically expressed in the egg cell prior to fertilization [29]. In *Hieracium*, most of the putative *EC1* orthologs were up-regulated in the pre-pollination sample of *lop138*, which suggests that in *Hieracium* these sequences may also have signaling roles prior to fertilization (Table 3). However, these sequences were also detected in apomictic R35 and A36, and although not identified as differentially expressed, most of the sequences showed expression in pre-embryo and post-embryo stages of R35 (Table 3). Similarly, orthologs of *RCD1*, which is involved in the onset of sexual reproduction through its differentiation controlling function in yeast [28], were observed to be up-regulated during seed development in *lop138*, R35 and A36. The expression patterns observed from *EC1* and *RCD1* orthologs during this study suggest that molecular signals marking sexual reproduction can be detected in apomictic ovules and that apomictic seed development might be using elements of the sexual pathway to promote autonomous seed development. This corresponds with the current understanding that the apomixis pathway is overlaid on the default sexual pathway and uses elements of sexual reproduction to initiate apospory and autonomous seed development in somatic cells [24].

Koltunow et al. [24] were able to demonstrate that meiosis in the MMC, which is an initiation of the sexual pathway, was necessary for initiation of the AIs, which are the origins of aposporous embryo sacs. They performed experiments that specifically hindered meiosis in the MMC and observed that AIs and the subsequent aposporous embryo sacs were not being formed, which suggested that the function of *LOA* was dependent on the initiation of the sexual pathway [24]. The *LOP* locus may be operating in a similar manner to the *LOA* locus in that it functions in response to cues of the sexual pathway. The identification of *Hieracium* sequences that are orthologous to genes involved in the initiation of sexual reproduction in other species, such as *EC1* and *RCD1,* is an important contribution towards understanding mechanisms of parthenogenesis in *Hieracium*.

Koltunow et al. [24] proposed that the two loci conferring apomixis in *Hieracium*, later determined to be *LOA* and *LOP*, silence sexuality epigenetically or genetically. A similar model was proposed for the apomixis-controlling region (ACR) in *Paspalum*. The ACR was characterized by high cytosine methylation and was located on heterochromatin knobs [52]. Podio et al. [52] hypothesized that the ACR contained parthenogenesis activator and suppressor genes and that cytosine methylation in ACR could be inactivating the parthenogenesis suppressor genes, allowing precocious parthenogenesis to occur. To test their hypothesis, Podio et al. [52] specifically inhibited DNA methyltransferases, in order to prevent methylation, and discovered that parthenogenesis was negatively affected. The effect of demethylation in *Paspalum* is similar to the effect of deletion in the *LOP* locus in *Hieracium*, as *LOP* loss-of-function mutants revert to sexuality [16]. This suggests that genetic components that are either involved in suppressing sexuality or in suppressing parthenogenesis-suppressor genes were lost in *lop* mutants.

In addition to epigenetic regulation, pathways involved in biotic and abiotic signaling are likely to be involved in the induction of autonomous seed development. In R35, transcripts putatively encoding proteins with roles in auxin transport, response to stress, stem cell niche specification and maintenance and embryo patterning were up-regulated in pre-embryo and post-embryo stages of R35 (Fig. 5). In *lop138*, these pathways were up-regulated post-pollination (Fig. 5). These observations indicated that apomictic seed development might be induced through the effects of many pathways involving environmental cues, such as transport of the growth hormone auxin, response to stress and signal transduction. These pathways likely recruit genes involved in cell fate determination during early ovule development, resulting in precocious embryogenesis. Other studies also linked auxin to somatic embryogenesis [53] and to autonomous seed coat development in addition to endosperm initiation post-pollination [54]. Stress was also proposed to induce somatic embryogenesis [55] and many studies observed a relationship between stress-inducing signaling pathways and somatic embryogenesis induction [56–58].

The transcriptome data was also used to identify transcripts marking stages undergoing aposporous embryo sac development and embryo development. By utilizing abundance estimates for the transcriptome, transcripts preferentially active during aposporous embryo sac development in R35, during stages with developing embryos in R35, A36 and *lop138* were identified (Fig. 7; Additional file 10). These sequences can serve as a short list for investigating the developmental stages they mark. The validity of the list of developmental marker genes is demonstrated by the representation of genes already known to be associated with the respective developmental events, such as *LOX2*, *AGL61* and *PHE1*. We identified an ortholog of *LOX2* as marking stages undergoing aposporous embryo sac development (Additional file 10 A), which is in agreement with previous studies that found *LOX2* to be associated with AI cell. The same ortholog was down-regulated in mutants with deletion in *LOA*, i.e. the locus responsible for AI initiation [51, 59]. *AGL61*, which is expressed specifically in the central cell of the female gametophyte and early endosperm in *Arabidopsis* [48, 60], was identified as a marker of stages undergoing embryo and endosperm development in the apomictic and the mutant accessions alike (Fig. 6). Similarly *PHE1*, which is a paternally expressed imprinted gene that is expressed in the embryo and endosperm of *Arabidopsis* [61, 62], was identified as marking stages undergoing embryo and endosperm development in the apomictic and the mutant accessions (Additional file 10 B).

Seed development in R35 and A36 is largely autonomous (no paternal input), thus expression of *PHE1*, which is supposed to be silenced when inherited maternally and expressed when inherited paternally in *Arabidopsis* [61, 62] suggests a modified imprinting system in R35 and A36. [63], suggested that in pseudogamous apomicts, where the canonical 2 m:1p genomic ratio of the endosperm is disrupted due to fertilization of two unreduced polar nuclei by a reduced sperm (4 m:1p genomic ratio), modification of the imprinting system could counteract the excess maternal genome in the endosperm. This could be achieved by reduced silencing of imprinted genes in the polar nuclei (paternalizing), thus increasing the dosage of paternally expressed imprinted genes [63]. Taken together, the identification of a transcriptionally active ortholog of a paternally expressed imprinted gene, *PHE1*, in both sexually derived seeds and apomictic seeds of *Hieracium* highlights that the imprinting system may be modified in *Hieracium*. Alternatively, imprinted genes of *Hieracium* may differ from those of *Arabidopsis*. Recent studies suggested that some imprinted genes might be species specific due to neofunctionalization of duplicated genes. For instance the FIS2-PRC2 complex is proposed to be Brasicaceae-specific and resulted from duplication of the VRN-PRC2 complex members that, in *Arabidopsis,* assumed a new role in gametophyte and seed development [64]. Genome duplication is a feature of the polyploid *Hieracium* species used in this study, therefore neofunctionalized imprinted genes could differ from those known in other species such as *Arabidopsis*. To simplify the analysis, duplicated transcripts were removed from the transcriptome, however, none of these transcripts were from PRC2 complex genes or genes involved in imprinting (Additional file 11).

The reference transcriptome is expected to be useful to further investigate the roles of PcG genes in *Hieracium*. Down-regulating members of the PcG complex that function to repress central cell proliferation prior to fertilization in *Arabidopsis*, such as *FIE*, did not result in autonomous endosperm development in sexual *Hieracium* [22, 23]. *FIE* was not linked to the *LOP* locus in *H. praealtum* [24]. However, it was still needed for autonomous seed development as its down-regulation in apomictic *Hieracium* species resulted in decreased initiation of autonomous seed development and increased seed abortion [23]. In this study, only one sequence encoding a protein with similarity to *Arabidopsis* FIE (AtFIE) was expressed in all stages of R35 (Table 4). This agrees with a previous observation that a single allele of *FIE* was expressed in ovaries of apomictic and sexual *Hieracium* [23]. Although *FIE* was necessary for seed development in both apomictic and sexual *Hieracium*, it did not interact with many members of the PcG complex, such as *MEA*, *MSI1* and *RBR* in vitro [23]. *Hieracium* proteins interacting with FIE are still unknown. [23] suggested SWINGER (EZI1) is likely to interact with FIE in *Hieracium* (HFIE), because it is a SET-domain containing PcG protein and shows higher homology to AtCLF than AtMEA. SWINGER’s homology to AtCLF is important as AtCLF was the only PcG protein HFIE interacted with in vitro [23]. Here, sequences encoding proteins with high similarity to SWINGER and AtCLF were identified (Table 4). These sequences provide resources for future studies investigating the role of *FIE* during seed development in *Hieracium*.

## Conclusions

Developing apomictic crop plants has the potential to revolutionise modern agriculture. It will provide a simple means to fix hybrid vigour by allowing clonal seed production from the high yielding individuals produced after crossing [2]. Farmers will then be able to collect and grow seed for the next season, enable efficient and consistent yields of high-quality seeds, fruits and vegetables at lower costs.

Our ultimate goal is to identify the genes conferring apomixis so that the trait can be engineered into crop species. To achieve this, we have been studying the model apomict *Hieracium* and have previously identified two loci (LOP and LOA) required for *Hieracium* to be an apomict. Mutant in the lop locus exhibits loss of parthenogenesis and autonomous endosperm development. The comprehensive floral reference transcriptome for *Hieracium* produced here provides a valuable resource for research into the molecular basis of apomixis and the identification of the genes underlying the LOP locus. Our results support the idea that apomictic and sexual seed developmental pathway share common components and that it is the switching on these pathways without the need for pollination that allows apomixis [15, 24]. We identify many genes that play important roles in reproductive development and potential marker genes for different stages of seed development. These genes provide a way to examine in more fine detail the transcriptional changes associated with the key apomictic events of apomeiosis, parthenogenesis and autonomous endosperm development.

In R35, parthenogenesis and autonomous endosperm development are controlled by the *LOP* locus and are inherited as a single unit. However, recombination is suppressed at the *LOP* locus, which made resolving the level of dependency between the genetic components of parthenogenesis and autonomous endosperm formation in R35 difficult [16]. A study involving other apomictic and sexual species of *Hieracium* showed that autonomous endosperm could be genetically separated from parthenogenesis, suggesting parthenogenesis and autonomous endosperm formation might be controlled by separate components of the *LOP* locus that are tightly linked in R35 [65]. The transcriptomic data produced for this study should contribute towards resolving the genetic components of the *LOP* locus. With the availability of gene models for *Hieracium* [51] and a substantial EST database and high-density genetic map of lettuce (*Lactuca sativa*) in Asteraceae [66], the RNA-seq data of R35 and its derivative mutant lop138 can be mapped to the genomic resources of *Hieracium* and lettuce. This transcriptome should help identify the genome-wide regulatory differences between the apomictic R35 and the mutant lop138 and the genetic components of the LOP loci involved in apomixis.

## Methods

### Plant material

Plant material was obtained from live specimens of wild type apomictic plants and a mutant plant (ϒ 138) derived from an apomictic accession [16]. The wild-type plant *Hieracium praealtum* (Vill.) Zahn is referred to by its accession code R35. For clarification, this plant was previously referred to as *Hieracium caespitosum* (C4D) [16] but has since been identified to be *H. praealtum*. *Hieracium aurantiacum* L. was used to pollinate the mutant accession and is referred by its accession code A36. The mutant accession ϒ138, referred to as ‘*lop138*’, was derived from R35 through gamma irradiation, which deleted the *LOSS OF PARTHENOGENESIS* (*LOP*) locus (Catanach, Erasmuson, Podivinsky, Jordan and Bicknell 2006). *lop138* retained the *LOSS OF APOMEIOSIS* (*LOA*) locus, therefore it produces aposporous embryo sacs but fails to initiate autonomous seed development (Catanach, Erasmuson, Podivinsky, Jordan and Bicknell 2006). Upon fertilization *lop138* produces viable seeds and hybrid progeny with increased ploidy level compared with that of the maternal parent (Koltunow, Johnson, Rodrigues, et al. 2011). R35, *lop138* and A36 were propagated in a heated glasshouse at the Department of Botany (University of Otago), in fertilized potting mix, under natural daylight augmented with SON-T AGRO 400w lights to provide 16 h of light per day.

### Screening embryo sacs for developing embryos

To determine the stages of the capitulum that contained ovules undergoing embryo development, groups of florets were removed from capitula at the stages described by Koltunow et al. [9] and secured on a microscope slide with double-sided tape. A drop of water was then placed on the florets to submerge them and under a dissecting microscope the pappus of each floret was peeled away, along with the ovary walls. This exposed the ovule. A slit was made in the middle section of the ovule integuments with fine-tipped forceps (size 5 Dunmont forceps), which allowed the contents of the ovule to float in the surrounding water. The embryo sac could then be detached from the ovule with the forceps. Each embryo sac was washed off the forceps with safranin staining solution onto a new microscope slide. This was done under a microscope to ensure that the embryo sac was dislodged. The embryo sac was then left in the staining solution for 5 min. After staining, the embryo sac was viewed under a compound microscope to discern the presence of the embryo.

### Procedure for tissue collections and RNA isolation

To collect ovaries at specific stages, a group of florets were removed from the appropriate stage of capitulum development with forceps and laid on a microscope slide. Ovaries were cut off with a clean RNase free razor blade then put into RNA extraction buffer. After collecting all ovaries from a capitulum, ovaries were homogenized for subsequent RNA extraction. This method was used for capitula from stages 5 to 10 of Koltunow et al. [9]. Florets of stages 3 and 4 were too small to grasp with forceps, therefore the bracts were removed from a capitulum. Then, while still attached to the receptacle, florets were put on a microscope slide. The vegetative parts of florets were cut off and removed from the slide. Ovaries were scraped off the receptacle and put into lysis buffer for RNA extraction. RNA was extracted with MagMAX™-96 Total RNA Isolation Kit (Ambion), which included a DNase step.

To collect ovule at specific stages, a floret was removed from the appropriate staged capitulum with forceps and attached to a microscope slide with double-sided tape as described above for staging. Then RNase free water was put on the floret to fully submerge it. Under a dissecting microscope the pappus was peeled away, along with the ovary walls. This exposed the ovule, which was removed with forceps and transferred into a tube containing storage medium. Multiple ovule material was combined and total RNA was extracted with RNAqueous®-Micro Kit (Ambion) which included a DNase step. The RNA samples and yields are summarized in (Additional file 1: Table S1).

### RNA sequencing

At the New Zealand Genomics Facility (NZGL) (University of Otago), TruSeq RNA Sample Prep Kits were used to prepare cDNA libraries from total RNA extracted from ovule and ovary tissue of R35, *lop138* and A36 (Table 1). RNA integrity was checked with the Agilent bioanalyzer 2100 and only RNA samples with RIN value over 7 were used for the cDNA preparation. A total of 18 cDNA libraries were prepared, eight of which were from ovary samples of R35 (not replicated) and five that were from ovule samples of R35, *lop138* and A36 with biological replication. These libraries were sequenced on an Illumina HiSeq2000, via the paired end sequencing method (100 bp), across four sequencing lanes. Quality control of the sequence reads was performed using FastQC (version 0.10.1) [67].

### De novo transcriptome assembly procedure

To determine the relative expression levels of specific genes across the range of developmental stages and genotypes sampled, we first produced a de novo assembled reference transcriptome using sequence data from our 18 cDNA libraries. To produce the reference transcriptome the following was performed: DynamicTrim was run on the FASTQ data with the ‘-h 20’ parameter which sets a quality score cutoff of 20. LengthSort was run on the trimmed data with the ‘-l 50’ parameter to select reads of at least 50 bases. The first 4,000,000 reads of each of the trimmed and sorted data files were combined into two separate files (one for each read1 and read2 of read pairs) and the Trinity assembler (version r2011-08-20) [68] was run on the resulting combined files. Alignment and generation of sam files was done using bwa aln and bwa sampe (0.6.1-r104).

### Assessment of assembly validity

The validity of the reference assembly was assessed using two methods:

1. The percent of reads that mapped back to the reference assembly (PMBR) was calculated for each library. This was done by extracting and counting mapped reads of each alignment file (.sam) using samtools and the bash command wc –l. Total sequenced reads were also similarly counted from each .sam file. The PMBR was then calculated as (mapped reads / total reads)*100.
2. All available expressed sequence tag (EST) sequences for *Hieracium* and other Asteraceae species were downloaded from NCBI (last accessed on 4th April 2014). Standalone BLAST (ncbi-blast-2.2.27+) [69] was used to perform BLASTN search of the newly reconstructed *Hieracium* transcriptome against these ESTs at an e-value cut-off of 1e-4.

### Assessment of completeness

The completeness of the transcriptome was assessed based on sequence contiguity (N50), which is the minimum assembly size in which at least 50% of the assembled bases are found, and by searching for Benchmarking Universal Single-copy Orthologs (BUSCOs), in the Eukaryotic set of BUSCOs downloaded from http://busco.ezlab.org/, and by searching the *Hieracium* transcriptome by using BUSCO_v1.1 software [70].

### Removal of chimera and redundant transcripts

A total of 1,165,822 plant protein reference sequences (RefSeq) were downloaded from NCBI (ftp://ftp.ncbi.nlm.nih.gov/refseq/release/) and used as a database for BLASTX (ncbi-blast-2.2.27+) [69] with an e-value cut-off of 1e-2. To remove chimeric transcripts, a pipeline and scripts by Yang and Smith [71] were obtained (https://bitbucket.org/yangya/optimize_assembler) and implemented. The results from the homology search were used to identify and remove chimeras from the transcriptome. To remove redundant transcripts, CD-HITS-EST version 4.5.4 [72, 73] was used to remove sequences that were entirely aligned to longer transcripts with a similarity threshold of 90%. The redundant transcripts that were differentially expressed but removed are provided in Additional file 11.

### Annotation

The annotation strategies are summarized in (Additional file 1: Table S10). To annotate the non-redundant transcriptome, two high-quality protein sequence databases were downloaded and formatted as BLAST databases. The two databases used the reference sequence database (RefSeq) containing protein sequences from plant species (downloaded from (ftp://ftp.ncbi.nlm.nih.gov/refseq/release/) and all protein sequences included in the Swiss-Prot section of UniProtKB (downloaded from http://www.uniprot.org/uniprot). BLAST analysis (ncbi-blast-2.2.27+) [69] was used to search the *Hieracium* transcripts in the two protein databases using the BLASTX algorithm at an e-value cutoff of 1e-4. The results from these searches were merged and consolidated to produce a final annotation for the transcriptome. If a *Hieracium* transcript got hits to entries from both RefSeq and Swiss-Prot, the Swiss-Prot result was chosen as Swiss-Prot is manually curated and often supported via experimental or literature evidence. To identify novel transcripts potentially encoding full-length peptides, transcripts without any BLAST hits to EST or protein databases, were screened for Open Reading Frames (ORFs) using the ORF-predictor server (http://proteomics.ysu.edu/tools/OrfPredictor.html). The resulting predicted peptides were filtered, using custom python and R Scripts (Additional file 12) to only retain transcripts with predicted peptides that are at least 100 amino acids long.

### Gene ontology term mapping

After annotating the transcriptome via BLASTX, IDs of loci encoding proteins that matched the predicted peptides encoded by the query sequence (subject IDs) were used to assign GO terms to the transcripts. To accomplish this, the subject IDs in the BLASTX result, which came from two different databases (Swiss-Prot and RefSeq), were first converted to entrez gene IDs using the following steps: 1) The Uniprot ‘Ac IDs’ were mapped to entrez gene IDs on the uniprot ID mapping service (http://www.uniprot.org/uploadlists/). 2) The RefSeq ‘gi IDs’ were first mapped to UniProtKB ‘AC IDs’ then the ‘AC IDs’ mapped to entrez gene IDs on the uniprot web site (http://www.uniprot.org/uploadlists/). Using a custom R script (File S1), GO terms were mapped to the gene IDs from an NCBI file (gene2go.gz) (ftp://ftp.ncbi.nlm.nih.gov/gene/DATA/, last accessed on 19th March 2015), which contains a comprehensive list of gene IDs and their corresponding GO IDs (File S2).

### Quantifying and normalizing expression

To estimate transcript abundance across the sampled conditions, the number of reads supporting each transcript was counted from the alignment file (.sam). The reads aligned to each transcript, with a minimum mapping quality (MAQ) of 1, were counted using the sam2count.py script [74]. The digital count data was then normalized using two different methods:

1. The Transcripts Per Million (TPM) method of normalization [75] was applied to correct for transcript length and sequencing depth effects. TPM is a slight modification of the popular RPKM method and it was calculated using the formula of [75] in a custom R script as follows; the normalization factor (z) was calculated by dividing each transcript’s read count (tc) by its length (tl) and summing these values for each library (z = sum(tc/tl)). The TPM for each transcript was then calculated by multiplying its read count by one million and dividing it by the product of its length and z (TPM = (tc*1e6)/(z*tl)).
2. The Universal exPression Code (UPC) method of normalization [49] was also used to scale the digital counts into values indicating the activity levels of genes from zero to one. The UPC package (version 2.10.8) was obtained from bioconductor (http://bioconductor.org) and the ‘UPC_RNASeq’ function was implemented with user defined “GC annotation” enabled. A custom python script (Additional file 12) was used to count GC content of each transcript, which was then used as “GC annotation” file in UPC_RNASeq.

Figures 2, 3, 4, 5 and 6 were created using TPM values that were median centered and hierarchically clustered using heatmap.2 function in R. For visualizing the expression of each transcript across the samples, the median centered and clustered expression values were scaled by z-score and attributed a color, with bright red representing highest expression and light green representing lowest expression.

## Additional files

Additional file 1: Additional Figures S1-S5 and Tables S1-S10. (DOCX 6791 kb)

Additional file 2: Differential expression analysis to discover changes associated with parthenogenesis in R35. (XLSX 256 kb)

Additional file 3: Differential expression analysis to discover changes associated with apomictic seed development in R35. (XLSX 1028 kb)

Additional file 4: Differential expression analysis to discover changes associated with sexually-induced seed development in lop138. (XLSX 2077 kb)

Additional file 5: Differential expression analysis to discover differences associated with quiescent embryo sac in *lop*138 relative to R35. (XLSX 2718 kb)

Additional file 6: GO term enrichment analysis in the differentially expressed transcripts resulting from comparing samples of apomictic accessions (R35 and A36) to samples of the mutant accession (*lop*138). (XLSX 4571 kb)

Additional file 7: Differentially expressed transcripts with no GO annotations and with or without BLAST hits. (XLSX 974 kb)

Additional file 8: Table of differentially expressed transcripts associated with “epigenetic regulation of gene expression”. (XLSX 15 kb)

Additional file 9: Transcripts showing stage-preferred expression. (XLSX 61 kb)

Additional file 10: List of genes marking stages undergoing aposporous embryo sac development in R35. (XLSX 17 kb)

Additional file 11: Transcripts that are differentially expressed but identified as duplicates and removed from transcriptome during processing. (XLSX 68 kb)

Additional file 12: R scripts. (DOCX 20 kb)
