# Supplementary material for: Comparative transcriptome analysis of the wild-type model apomict Hieracium praealtum and its loss of parthenogenesis (lop) mutant
Source: BMC Plant Biol. 2018 Sep 24;18:206. doi: 10.1186/s12870-018-1423-1 (PMC6154955; doi:10.1186/s12870-018-1423-1)
Supplement: Supplementary file 1 — Additional Figures S1-S5 and Tables S1-S10. (DOCX 6791 kb) [file 12870_2018_1423_MOESM1_ESM.docx]

Additional File 1. Figures 1-5 & Tables 1-10

**Comparative transcriptome analysis of the wild-type model apomict *Hieracium* *praealtum* and its *loss of parthenogenesis* (*lop*) mutant**

**Sophia Bräuning, Andrew Catanach, Janice Lord, Ross Bicknell and Richard C. Macknight^*^**

*** Correspondence:** email: [richard.macknight@otago.ac.nz](mailto:richard.macknight@otago.ac.nz)

Additional File 1 (Fig 1). Developmental stages of *Hieracium*. (A) Capitulum development from stage 1 to stage 14 (10), adapted from Koltunow et al. 2011a. Stages 1 - 5 are enclosed in bracts, at stage 6 the bracts open at the tip, stages 7 – 9 bracts are fully open and the florets open starting at the outer whorl, from stage 10 – 13 the florets senesce and the developing seeds are enclosed in the bracts. At stage 14 the seeds are mature and bracts open again. A single floret is shown between stages 9 and 10 (scale bar is 500 μm). Images B – D are of manually dissected embryo sacs, arrows pointing to embryos; (B) *Hieracium* *praealtum* (R35) stage 7, (C) *Hieracium* *praealtum* lop mutant (*lop138*) stage 12 (D) *Hieracium aurantiacum* stage 10.

Additional File 1 (Fig 2). Enriched GO categories for DE set of R35 associated with initiation of autonomous seed development.

In order to identify DE transcripts associated with initiation of autonomous seed development, R35 samples obtained from ovules carrying mature embryo sacs (Wt_E) were compared to R35 samples obtained from ovules undergoing parthenogenesis (Wt_L). These DE transcripts were analyzed to identify enriched GO terms. Percent in background = number of transcripts in GO category/ total number of annotated transcripts (40576), percent in DE = number of DE transcripts in GO category/ total number of annotated DE transcripts (333).

Additional File 1 (Fig 3). Enriched GO categories for DE set of R35 associated with seed development.

In order to identify DE transcripts associated with apomictic seed development, R35 samples obtained from ovaries and ovules carrying mature embryo sacs (Ovary_5 & Wt_E) were compared to R35 samples obtained from ovaries carrying developing embryos (Ovary_7 – Ovary_10). These DE transcripts were analyzed to identify enriched GO terms. Percent in background = number of transcripts in GO category/ total number of annotated transcripts (40576), percent in DE = number of DE transcripts in GO category/ total number of annotated DE transcripts (1071).

Additional File 1 (Fig 4). Enriched GO categories for DE set of *lop138* associated with fertilization-induced seed development.

In order to identify DE transcripts associated with fertilization-induced seed development in *H. praealtum*, *lop138* samples obtained from ovules carrying mature embryo sacs (lop_E) were compared to *lop138* samples obtained from ovules carrying developing embryos (lop_L). These DE transcripts were analyzed to identify enriched GO terms. Percent in background = number of transcripts in GO category/ total number of annotated transcripts (40576), percent in DE = number of DE transcripts in GO category/ total number of annotated DE transcripts (2400).

Additional File 1 (Fig 5). Enriched GO categories for DE set resulting from comparing *lop138* to apomictic accessions R35 and A36.

In order to identify differences between apomictic *Hieracium* and the *Hieracium* plant incapable of autonomous seed development, *lop138*, each *lop138* sample (pre-pollination and post-pollination) was compared to each R35 ovule sample (pre-embryo and post-embryo) and the post-embryo sample of A36. Percent in background = number of transcripts in GO category/ total number of annotated transcripts (40576), percent in DE = number of DE transcripts in GO category/ total number of annotated DE transcripts (5631).

**Tables**

**Additional File 1 (Table S1).** **Total RNA yield per sample.**

| **Sample ID** | **Number of Ovules** | **RIN^#^** | **Total RNA in micrograms** |
| --- | --- | --- | --- |
| Wt_E1 | 89 | 8.7 | 1.06 |
| Wt_E2 | 92 | 9.2 | 1.55 |
| Wt_L1 | 50 | 9.3 | 1.85 |
| Wt_L2 | 50 | 9.3 | 1.74 |
| lop_E1 | 89 | 9.3 | 2.05 |
| lop_E2 | 93 | 8.7 | 2.85 |
| lop_L1 | 50 | 8.0 | 2.08 |
| lop_L2 | 50 | 9.2 | 2.9 |
| Pol_L1 | 50 | 8.5 | 2.64 |
| Pol_L2 | 50 | 9.4 | 3.28 |
| Ovary_3 | 100 | 8.5 | 3.98 |
| Ovary_4 | 50 | 9.0 | 1.38 |
| Ovary_5 | 50 | 8.0 | 1.53 |
| Ovary_6 | 50 | 9.0 | 1.78 |
| Ovary_7 | 50 | 8.2 | 1.18 |
| Ovary_8 | 50 | 7.3 | 2.74 |
| Ovary_9 | 50 | 9.6 | 1.43 |
| Ovary_10 | 50 | 7.5 | 4.56 |

^#^ RIN is the RNA integrity measure produced by the bioanalyzer.

Additional File 1 (Table S2). Pairwise Spearman’s correlation coefficients of observed expression in samples.

Additional File (Table S3). Numbers of differentially expressed transcripts^#^ in each pairwise comparison of R35, *lop138* and A36 samples.

|  | **Wt_L**  **(R35 early embryogenesis)** | **Ovary_7 – Ovary_10 combined**  **(R35 post-embryo)** | **lop_E**  **(*lop138* pre-embryo**) | **lop_L**  **(*lop138* post-embryo)** |
| --- | --- | --- | --- | --- |
| Wt_E  (R35 pre-embryo) | 772  (565, 207) | 2196  (1715, 481) | 1729  (913, 816) | 8595  (5736, 2859) |
| Wt_L  (R35 early embryogenesis) |  |  | 708  (298, 410) | 4510  (3378, 1132) |
| lop_E  (*lop138* pre-embryo) |  |  |  | 5745  (4289, 1456) |
| Pol_L  (A36 post-embryo) |  |  | 4944  (3024, 1920) | 3718  (3013, 707) |

# The p values were adjusted for multiple testing with the Benjamini-Hocberg method, and an adjusted p value of 0.1 was used as cutoff. Numbers in brackets indicate up-regulated and down-regulated transcripts respectively.

Additional File (Table S4) Summary of number of DE transcripts without GO annotation and with or without BLAST hits.

|  | Number of transcripts DE in R35 not DE in lop138 | Number of Shared DE transcripts I R35 and lop138 | Number of transcripts DE in lop138 not DE in R35 |
| --- | --- | --- | --- |
| BLAST hit but no GO annotation | 621  Additional file 7A | 334  Additional file 7C | 1841  Additional file 7E |
| No BLAST or GO annotation | 396  Additional file 7B | 158  Additional file 7D | 1056  Additional file 7F |

Additional File 1 (Table S5). BLASTX result information of DE transcripts predicted to encode AGL61, AGL62 and AGL80.

| **Transcript ID** | **BLASTX top hits** | **% identity** | **e-value** |
| --- | --- | --- | --- |
| comp25008_c0_seq1 | Agamous-like MADS-box protein AGL61 | 36.71 | 5.00E-22 |
| comp30010_c0_seq1 | Agamous-like MADS-box protein AGL61 | 30.43 | 3.00E-11 |
| comp10709_c0_seq4 | Agamous-like MADS-box protein AGL62 | 67.65 | 2.00E-27 |
| comp22232_c0_seq1 | Agamous-like MADS-box protein AGL62 | 59.84 | 5.00E-46 |
| comp26433_c0_seq3 | Agamous-like MADS-box protein AGL62 | 63.27 | 2.00E-14 |
| comp30373_c0_seq1 | Agamous-like MADS-box protein AGL62 | 52.78 | 1.00E-08 |
| comp14184_c0_seq1 | Agamous-like MADS-box protein AGL80 | 57.69 | 8.00E-28 |
| comp9168_c0_seq1 | Agamous-like MADS-box protein AGL80 | 41.4 | 6.00E-30 |

Additional File 1 (Table S6). Pathways enriched in the gene sets showing capitulum stage-enhanced activity in R35.

| **Term** | **ID** | **p.value** | **Stage** |
| --- | --- | --- | --- |
| Indole-3-acetyl-amide conjugate biosynthesis | PWY-6219 | 0.05 | 3 |
| IAA degradation V | PWY-5788 | 0.05 | 3 |
| IAA degradation IV | PWY-2021 | 0.05 | 3 |
| (1,4)-beta-xylan degradation | PWY-6717 | 0.01 | 6 |
| UDP-D-xylose and UDP-D-glucuronate biosynthesis | PWY-4821 | 0.04 | 6 |

Additional File 1 (Table S7). Transcripts marking aposporous embryo sac development in R35.

The transcripts were identified based on their active status (UPC > 0.5) only in stages undergoing megagametogenesis in R35.

| **Active in stages of R35** | **Transcript Id** | **Transcript length in bp** | **Blastx top hit** | **description** | **% similarity** | **e-value** |
| --- | --- | --- | --- | --- | --- | --- |
| 3, 4, 5 | comp51990_c0_seq1 | 562 | XP_002263563.1 | uncharacterized protein LOC100244079 | 56.52 | 2.00E-46 |
| 3, 4 | comp10436_c0_seq1 | 702 | NLTP6_AMBAR | Non-specific lipid-transfer protein | 47.76 | 1.00E-13 |
| 3, 4 | comp22051_c0_seq1 | 785 | LOX21_SOLTU | Linoleate 13S-lipoxygenase 2-1, chloroplastic | 58.46 | 7.00E-103 |
| 3, 4, 5 | comp38300_c0_seq1 | 404 | GAT18_ARATH | GATA transcription factor 18 | 47.13 | 6.00E-34 |
| 3, 4 | comp5897_c0_seq1 | 678 | NLTP_PRUAV | Non-specific lipid-transfer protein | 52.17 | 5.00E-27 |
| 3, 4, 5, 6 | comp16071_c0_seq1 | 594 | ADS1_ARATH | Delta-9 acyl-lipid desaturase 1 | 53.63 | 2.00E-64 |
| 3, 4, 5, 6 | comp34347_c0_seq1 | 693 | IAA32_ARATH | Auxin-responsive protein IAA32 | 44.44 | 2.00E-40 |
| 3, 4, 5, 6 | comp34763_c0_seq1 | 704 | XP_007031706.1 | Transcription factor bHLH61 isoform 1 | 69.23 | 1.00E-62 |
| 3, 4, 5, 6 | comp53598_c0_seq1 | 534 | ALMT9_ARATH | Aluminum-activated malate transporter 9 | 50.86 | 4.00E-34 |

Additional File 1 (Table S8). Transcripts marking embryo development in R35, lop138 and A36.

The transcripts were identified based on their active status (UPC > 0.5) only in post-embryo stages of R35, *lop138* and A36.

|  | | | | | |
| --- | --- | --- | --- | --- | --- |
| **Transcript ID** | **Transcript length in bp** | **Blastx top hit** | **description** | **% similarity** | **e-value** |
| comp26069_c0_seq1 | 500 | XP_002527514.1 | conserved hypothetical protein | 48.94 | 2.00E-18 |
| comp8647_c1_seq1 | 788 | XP_004237665.1 | PREDICTED: uncharacterized protein LOC101251279 | 62.89 | 1.00E-31 |
| comp8834_c0_seq1 | 431 | XP_006284649.1 | hypothetical protein CARUB_v10005906mg | 50 | 2.00E-08 |
| comp35437_c0_seq1 | 1426 | XP_006429696.1 | hypothetical protein CICLE_v10011757mg | 56 | 9.00E-114 |
| comp75622_c0_seq1 | 376 | XP_007044810.1 | DORNROSCHEN-like, putative | 30.3 | 4.00E-07 |
| comp37269_c0_seq1 | 617 | XP_007216132.1 | hypothetical protein PRUPE_ppa019054mg, partial | 42.67 | 6.00E-08 |
| comp32167_c0_seq1 | 1163 | S47A2_RABIT | Multidrug and toxin extrusion protein 2 | 35.66 | 3.00E-44 |
| comp31559_c0_seq1 | 641 | PHE1_ARATH | MADS-box transcription factor PHERES 1 | 29.93 | 6.00E-15 |
| comp27961_c0_seq1 | 416 | C77A2_SOLME | Cytochrome P450 77A2 | 77.54 | 3.00E-74 |
| comp33940_c0_seq1 | 707 | C77A2_SOLME | Cytochrome P450 77A2 | 73.19 | 1.00E-111 |
| comp22321_c0_seq1 | 1167 | ARR12_ARATH | Two-component response regulator ARR12 | 35.48 | 4.00E-26 |
| comp42469_c0_seq1 | 1151 | AAP6_ARATH | Amino acid permease 6 | 64.09 | 3.00E-143 |
| comp57352_c0_seq1 | 511 | AAP6_ARATH | Amino acid permease 6 | 58.39 | 4.00E-46 |
| comp5831_c0_seq1 | 599 | C71A8_MENPI | Cytochrome P450 71A8 | 29 | 2.00E-20 |
| comp30010_c0_seq1 | 570 | AGL61_ARATH | Agamous-like MADS-box protein AGL61 | 30.43 | 3.00E-11 |
| comp34393_c0_seq1 | 986 | WRI1_ARATH | Ethylene-responsive transcription factor WRI1 | 76.12 | 1.00E-45 |
| comp18275_c0_seq1 | 804 | NFYB6_ARATH | Nuclear transcription factor Y subunit B-6 | 70.32 | 2.00E-60 |
| comp25118_c0_seq1 | 666 | ENT8_ARATH | Equilibrative nucleotide transporter 8 | 46.64 | 9.00E-55 |
| comp38502_c0_seq1 | 402 | LOG7_ARATH | Cytokinin riboside 5-monophosphate phosphoribohydrolase LOG7 | 92.86 | 7.00E-47 |
| comp8826_c0_seq1 | 831 | SCP28_ARATH | Serine carboxypeptidase-like 28 | 61.81 | 1.00E-73 |
| comp18211_c0_seq1 | 857 | PER11_ARATH | Peroxidase 11 | 66.23 | 7.00E-106 |
| comp47282_c0_seq1 | 797 | FBD2_ARATH | Putative FBD-associated F-box protein At1g55030 | 26.01 | 7.00E-08 |
| comp36994_c0_seq1 | 1297 | JOIN_SOLLC | MADS-box protein JOINTLESS | 61.76 | 1.00E-76 |
| comp30872_c0_seq1 | 712 | HDG11_ARATH | Homeobox-leucine zipper protein HDG11 | 53.81 | 2.00E-68 |
| comp72586_c0_seq1 | 386 | PABP8_ARATH | Polyadenylate-binding protein 8 | 47.92 | 9.00E-09 |
| comp21663_c0_seq1 | 685 | Y5161_ARATH | Uncharacterized protein At5g01610 | 27.62 | 5.00E-05 |
| comp18375_c0_seq1 | 754 | FBL77_ARATH | F-box/LRR-repeat protein At4g29420 | 29.51 | 3.00E-34 |
| comp30883_c0_seq1 | 1072 | APL_ARATH | Myb family transcription factor APL | 39.02 | 2.00E-09 |
| comp14128_c0_seq1 | 1281 | GDL29_ARATH | GDSL esterase/lipase At1g71691 | 70.41 | 1.00E-177 |
| comp34266_c0_seq1 | 454 | NFYB9_ARATH | Nuclear transcription factor Y subunit B-9 | 72.97 | 2.00E-54 |

Additional File 1 (Table S9). Transcripts marking R35 but not lop138.

The transcripts were selected based on their active status (UPC > 0.5) in any stage of R35 but inactive status (UPC < 0.5) in *lop138* pre-pollination sample.

|  | | | | | | |
| --- | --- | --- | --- | --- | --- | --- |
| **Active in lop138** | **transcript** | **Transcript length in bp** | Blastx top hit | **description** | **% similarity** | **e-value** |
| no | comp49361_c0_seq1 | 439 | XP_006357572.1 | PREDICTED: uncharacterized protein LOC102599307 | 47.69 | 2.00E-12 |
| no | comp60140_c0_seq1 | 438 | XP_008448008.1 | PREDICTED: retrovirus-related Pol polyprotein from transposon TNT 1-94 isoform X3 | 36.5 | 1.00E-19 |
| no | comp44797_c0_seq1 | 566 | MLO5_ARATH | MLO-like protein 5 | 55.61 | 1.00E-57 |
| no | comp44804_c0_seq1 | 474 | 7SB1_SOYBN | Basic 7S globulin | 32.35 | 3.00E-16 |
| no | comp54612_c0_seq1 | 457 | POL2_DROME | Retrovirus-related Pol polyprotein from transposon 297 | 42.86 | 1.00E-14 |
| no | comp8984_c0_seq1 | 1323 | PPO_MALDO | Polyphenol oxidase, chloroplastic | 51.94 | 2.00E-122 |
| no | comp60821_c0_seq1 | 419 | CUCM1_CUCME | Cucumisin | 52.7 | 5.00E-44 |
| no | comp23187_c0_seq1 | 584 | HDT1_SOLCH | Histone deacetylase HDT1 | 35.96 | 3.00E-14 |
| no | comp18435_c0_seq1 | 1290 | APO2_ARATH | APO protein 2, chloroplastic | 63.01 | 2.00E-65 |
| no | comp27405_c0_seq1 | 411 | APO2_ARATH | APO protein 2, chloroplastic | 83.33 | 4.00E-23 |
| no | comp24643_c0_seq1 | 978 | COL2_ARATH | Zinc finger protein CONSTANS-LIKE 2 | 45.63 | 7.00E-60 |
| Post-pollination | comp8044_c0_seq1 | 435 | FPF1_ARATH | Flowering-promoting factor 1 | 61.02 | 3.00E-16 |
| Post-pollination | comp5891_c0_seq1 | 370 | NLTP2_PRUAR | Non-specific lipid-transfer protein 2 | 71.43 | 3.00E-11 |
| Post-pollination | comp23758_c0_seq1 | 506 | Y6944_ORYSJ | B3 domain-containing protein Os06g0194400 | 45.11 | 5.00E-21 |
| Post-pollination | comp30513_c0_seq1 | 1147 | TPPH_ARATH | Probable trehalose-phosphate phosphatase H | 81.94 | 9.00E-126 |
| Post-pollination | comp69089_c0_seq1 | 403 | GORK_ARATH | Potassium channel GORK | 66.22 | 2.00E-29 |
| Post-pollination | comp7234_c0_seq1 | 877 | C86B1_ARATH | Cytochrome P450 86B1 | 72.73 | 9.00E-102 |
| Post-pollination | comp30246_c0_seq1 | 876 | LHTL8_ARATH | Lysine histidine transporter-like 8 | 94.16 | 0 |
| Post-pollination | comp30246_c0_seq2 | 533 | LHTL8_ARATH | Lysine histidine transporter-like 8 | 84.02 | 6.00E-84 |

**Additional File 1 (Table S10). Summary of annotation strategies**.

| **Annotation** | **Method** | **Considerations** | **Program / resource** | **Good result criteria** |
| --- | --- | --- | --- | --- |
| Linking transcripts to biological function | Based on sequence similarity to known genes or proteins | Type of sequences being compared to find similarity | BLASTN: when comparing DNA to DNA sequences | At least 70% identity over more than 100 bases. Or e-value lower than 1e-4 (Claverie and Notredame 2003) |
|  |  |  | BLASTX: when comparing DNA sequences to protein sequences | At least 25% similarity over 100 amino acids or e-value lower than 1e-4 (Claverie and Notredame 2003) |
|  | Based on Gene Ontology (GO) terms associated to genes and their products in a species-independent manner | Gene ontology aspects or categories; Biological process, Molecular function and Cellular component (Ashburner et al. 2000) |  |  |
| Linking transcripts to their expression dynamics | Based on the number of reads mapped to a transcript under each condition. | Mapping errors and ambiguity | Mapping Quality score (MAQ) (Li et al. 2008) | MAQ of 0 = read mappable to multiple locations  MAQ over 0 = read uniquely mappable |
|  |  | Biases associated with gene length, sequencing depth, GC content and Sequencing technology | Normalization methods such as, TPM (Wagner et al. 2012), RPKM (Mortazavi et al. 2008) and UPC (Piccolo et al. 2013) |  |
