# Supplementary material for: Comparative transcriptome analysis of the wild-type model apomict Hieracium praealtum and its loss of parthenogenesis (lop) mutant
Source: BMC Plant Biol. 2018 Sep 24;18:206. doi: 10.1186/s12870-018-1423-1 (PMC6154955; doi:10.1186/s12870-018-1423-1)
Supplement: Supplementary file 12 — R scripts. (DOCX 20 kb) [file 12870_2018_1423_MOESM12_ESM.docx]

Additional File 12. R scripts

**Comparative transcriptome analysis of the wild-type model apomict *Hieracium* *praealtum* and its *loss of parthenogenesis* (*lop*) mutant**

**Sophia Bräuning, Andrew Catanach, Janice Lord, Ross Bicknell and Richard C. Macknight^*^**

*** Correspondence:** email: [richard.macknight@otago.ac.nz](mailto:richard.macknight@otago.ac.nz)

# I. Scripts for filtering predicted peptides of transcripts without BLASTX hits.

Step 1: Obtain predicted peptide for transcripts from the ORF-predictor server (<http://proteomics.ysu.edu/tools/OrfPredictor.html>, accessed on 31 March 2015). This will produce a FASTA file, ‘file1.fasta’ in the script below.

Step 2: Use the following python script to count length of each predicted peptide. This will output a tab delimited text file (file2.txt) containing two columns; the first column will contain the transcript IDs and the second column will contain the length of the predicted peptides.

sequence = "file1.fasta"

fh = open(sequence)

dic = {}

out = "file2.txt"

ofh = open(out,'w')

for line in fh.readlines():

line = line.strip()

if line.startswith(">"):

count = 0

line = line.split()

id = line[0]

id = id[1:]

else:

for i in line:

count = count+1

dic[id]= count

print >> ofh, '\n'.join('{}\t{}'.format(key, val) for key, val in dic.items())

fh.close()

Step 3: Use the following R script to filter predicted peptides to keep those longer than 100 amino acids. The input files of this script are ‘file1.fasta’ and ‘file2.txt’ described in steps 1 and 2 above. The script below will output a FASTA file (‘file3.fasta’) containing transcripts that have predicted peptides longer than 100 amino acids.

pep_length = read.table("file2.txt", sep="\t")

dim(pep_length[pep_length$V2 >=100,])

pep_longer100 = pep_length[pep_length$V2 >=100,]

library("seqinr")

pred_pep = read.fasta(file="file1.fasta", as.string = TRUE, seqtype="AA")

longPep_fa = pred_pep[names(pred_pep)%in%pep_longer100$V1]

length(longPep_fa)

write.fasta(sequences=longPep_fa, names= names(longPep_fa), file.out = "file3.fasta")

# II. Scripts for mapping GO terms to transcripts with BLASTX hits.

Step 1: Obtain the file (gene2go.gz), which contains GO terms corresponding to gene identifiers from (ftp://ftp.ncbi.nlm.nih.gov/gene/DATA/, last accessed on 19^th^ march 2015).

Step 2: Follow steps 1 and 2 in section 3.2.9 of this thesis to convert Uniprot and RefSeq identifiers to entrez gene identifiers. The following tab delimited files will be produced from this step;

- “**UniprotACid.txt**” will have transcript IDs in the first column, the second column will have the top BLASTX hit for the transcript from Uniprot.
- “**UniprotAC_entrez.tab**” will have the Uniprot accession ID in the first column (column 2 in UniprotACid.txt) and the second column will have the corresponding entrez gene ID.
- “**RefSeq_gi.txt**” will have transcript IDs in the first column, the second column will have the top BLASTX hit for the transcript from RefSeq.
- “**RefSeq_gi_uniprotAC.txt**” will have RefSeq ID in the first column (column 2 of RefSeq_gi.txt) and the second column will contain corresponding Uniprot accession ID.
- “**RefSeq_gi_entrez.tab**” will have the Uniprot accession ID in the first column (column 2 in RefSeq_gi_uniprotAC.txt) and the second column will contain the corresponding entrez gene ID.

Step 3: Use the following R script to map GO terms to files produced in step 2 above.

# read in transcript id and respective Uniprot accession ID or RefSeq gi ids.

ac = read.table("UniprotACid.txt", header = T, sep = "\t")

gi = read.table("RefSeq_gi.txt", header = T, sep = "\t")

#read in entrez gene id mapped files

ac2geneid = read.table("UniprotAC_entrez.tab", header = T)

gi2uniprotac = read.table("RefSeq_gi_uniprotAC.txt", sep = "\t", header = T)

gi2geneid = read.table("RefSeq_gi_entrez.tab", header = T)

# merge ac (containing transcript id) with ac2gene id.

colnames(ac2geneid)[1]= "subjectid"

colnames(ac2geneid)[2] = "GeneId"

tran2ac2geneid = merge(ac, ac2geneid, by ="subjectid")

# merge gi to gi2uniprot to gi2gene

colnames(gi2uniprotac)[1] = "subjectid"

colnames(gi2uniprotac)[2] = "AC"

tran2gi2acc = merge(gi, gi2uniprotac, by = "subjectid")

colnames(gi2geneid)[1]= "AC"

colnames(gi2geneid)[2] = "GeneId"

tran2gi2ac2geneid = merge(tran2gi2acc, gi2geneid, by = "AC")

# now merge the two gene id containing objects. One has gi as 4th column the other only has 3 columns, so paste a column "no_gi" to make equal number of columns for rbinding

colnames(tran2ac2geneid)[1] = "Uniprot_AC"

colnames(tran2gi2ac2geneid)[1] = "Uniprot_AC"

colnames(tran2gi2ac2geneid)[2] = "gi"

tran2ac2geneid$gi = "no_gi"

tran2ac2geneid = tran2ac2geneid[,c(1,4,2,3)]

tran2geneid_combo = rbind(tran2ac2geneid,tran2gi2ac2geneid)

# now read in gene2go for mapping GO terms

gene2go = read.table("gene2go_march2015", header=T, sep="\t")

colnames(tran2geneid_combo)[4] = "GeneID"

tran2GO =merge(tran2geneid_combo, gene2go, by = "GeneID")

# save GO annotated sequences

write.table(tran2GO, "GO_annotated_transcripts.txt", quote=F, row.names=F, sep ="\t")

# III. Script for counting GC content of transcripts.

Step 1: Obtain the FASTA file containing transcript sequences.

Step 2: Use the following python script to count GC content of each transcript in the FASTA file. Input file = Trinity.Hieracium.fasta, output file = GC_count.txt

sequence = "./Trinity.Hieracium.fasta"

fh = open(sequence)

dic = {}

for line in fh.readlines():

line = line.strip()

if line.startswith(">"):

count = 0

line = line.split()

id = line[0]

id = id[1:]

else:

for i in line:

if i == "C" or i == "G":

count = count+1

dic[id]= count

fh.close()

length = "./Transcript_length.txt"

cf = open(length)

out = "./GC_count.txt"

ofh = open(out,'w')

line = cf.readline()

while line:

line = line.strip()

line = line.split("\t")

tran = line[1]

if tran in dic:

print >> ofh, tran,line[0],dic[tran]

line = cf.readline()

cf.close()

ofh.close()
